# Supplementary material for: Cost-effectiveness of a patient-reported outcome-based remote monitoring and alert intervention for early detection of critical recovery after joint replacement: A randomised controlled trial
Source: PLoS Med. 2024 Oct 9;21(10):e1004459. doi: 10.1371/journal.pmed.1004459 (PMC11463742; doi:10.1371/journal.pmed.1004459)
Supplement: S2 Appendix — (PDF) [file pmed.1004459.s002.pdf]

# Short description of the use of heartbeat ONE along the PROMoting Quality patient pathway

## 1. During Hospital Stay

At hospital admission, the study nurse creates the patient in heartbeat ONE and initiates the baseline PROM questionnaire. The patients completes the survey via the patient interface.

At patient discharge, the study nurse triggers the discharge PROM questionnaire. The patients completes the survey via the patient interface. After survey completion, the software randomly assigns the patient to the control or intervention group.

## 2. Documentation

After patient discharge, the study nurse uses the documentation template form provided in heartbeat ONE to document the predefined clinical parameters of the patient.

## 3. PROM-based remote Monitoring

A patient of the intervention group receives PROM questionnaires 1, 3 and 6 months after surgery. The questionnaires are provided via automatic email follow-ups, that contain a link to the browser-based patient interface.

Automated digital alerts signal a critical recovery path to the study nurse.

All patients receive a final PROM questionnaire via email followup 12 months after discharge.

# User interface for patients

**Tablet app within the clinic**

heartbeat collects all the necessary information so that doctors can check the patient‘s health status before taking a medical history.

- Minimal, patient-friendly design
- Collection of medical history and PROMs before treatment
- Integration with hospital information systems

**Web interface outside the clinic**

With automated follow-up of patients via email, our system enables doctors to learn from their treatments. The patients are asked about their state of health at home at predefined intervals.

- Preconfigured and automated follow-up questionnaires and reminders
- Secure, encrypted web interface
- No installation of an app required

## User interface for doctors

**Intelligent data acquisition**

- Our user interface enables the physician to receive a clear picture of the status of data collection and provides the necessary measures to plan the treatment steps.
- PROM data acquisition is preconfigured and reminders are sent automatically.
- PROM questionnaires are calculated automatically.

**Outcomes dashboards & PROMs-alerting**

- heartbeat offers a dashboard where real-time results at the individual and population level are visible.
- The dashboards enable the treatment team to take action immediately based on results and alerts.
- Benchmarking and comparisons at group level.

**Standardized sets**

- heartbeat offers standardized outcomes sets that are tailored to care pathway to serve as a robust basis for comparisons.
- Outcome sets include a wide range of questionnaires to the highest standard and scientifically validated PROMs.

**Compatibility**

- A bidirectional interface enables data exchange with electronic health records, to connect clinical data and PROMs.
- Compliant with the following interoperability standards: HL7 V2 and xDT.
