# Supplementary figures and images for: Cost-effectiveness of a patient-reported outcome-based remote monitoring and alert intervention for early detection of critical recovery after joint replacement: A randomised controlled trial
Source: PLoS Med. 2024 Oct 9;21(10):e1004459. doi: 10.1371/journal.pmed.1004459 (PMC11463742; doi:10.1371/journal.pmed.1004459)

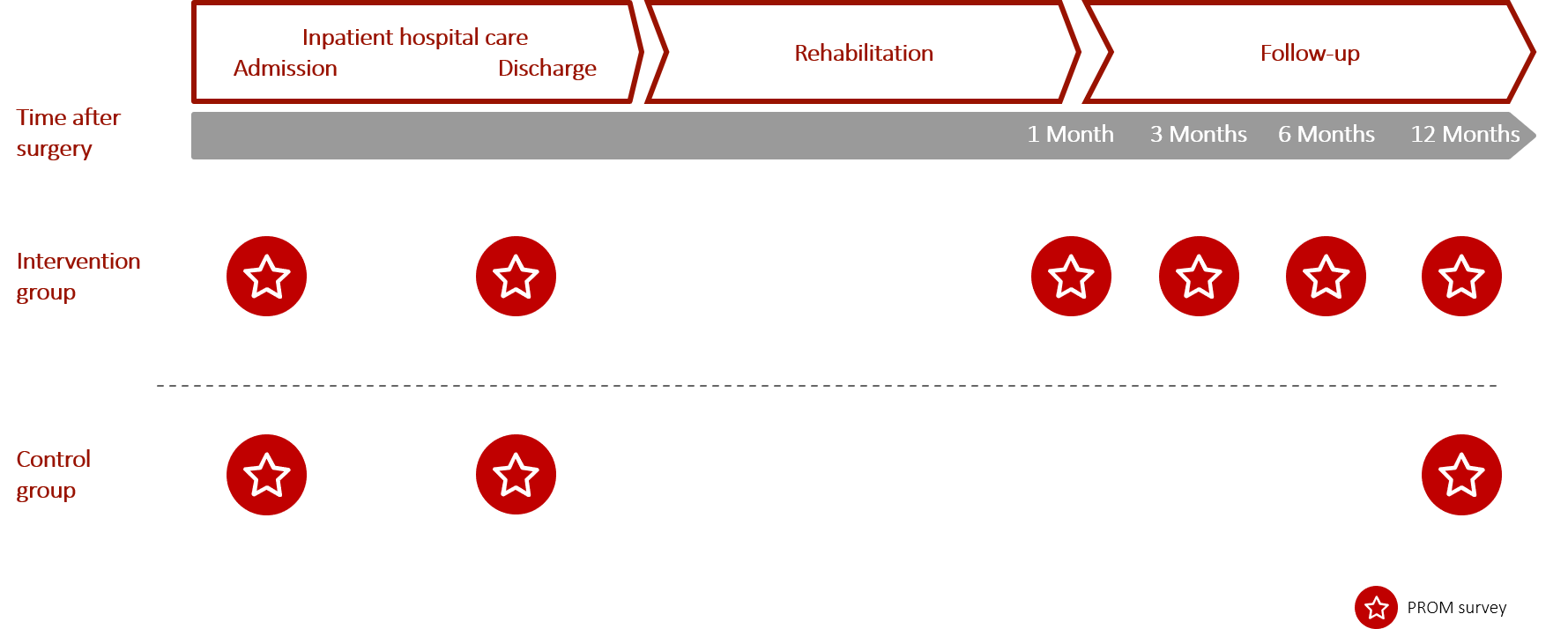

Supplement: S1 Fig — (TIF) [file pmed.1004459.s004.tif]

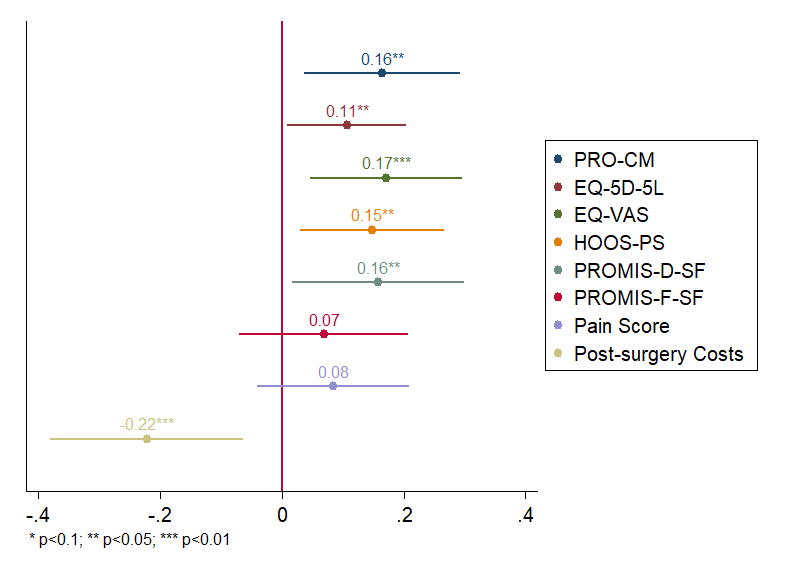

Supplement: S2 Fig — (TIF) [file pmed.1004459.s005.tif]

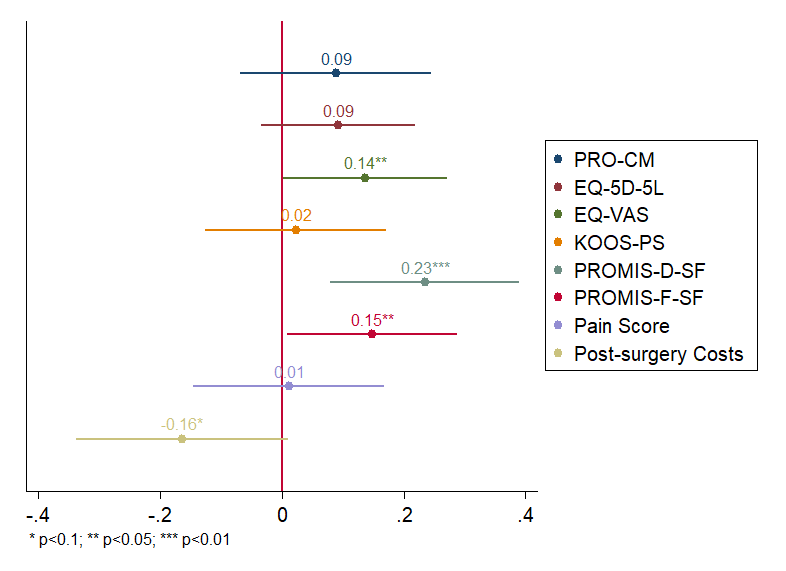

Supplement: S3 Fig — (TIF) [file pmed.1004459.s006.tif]

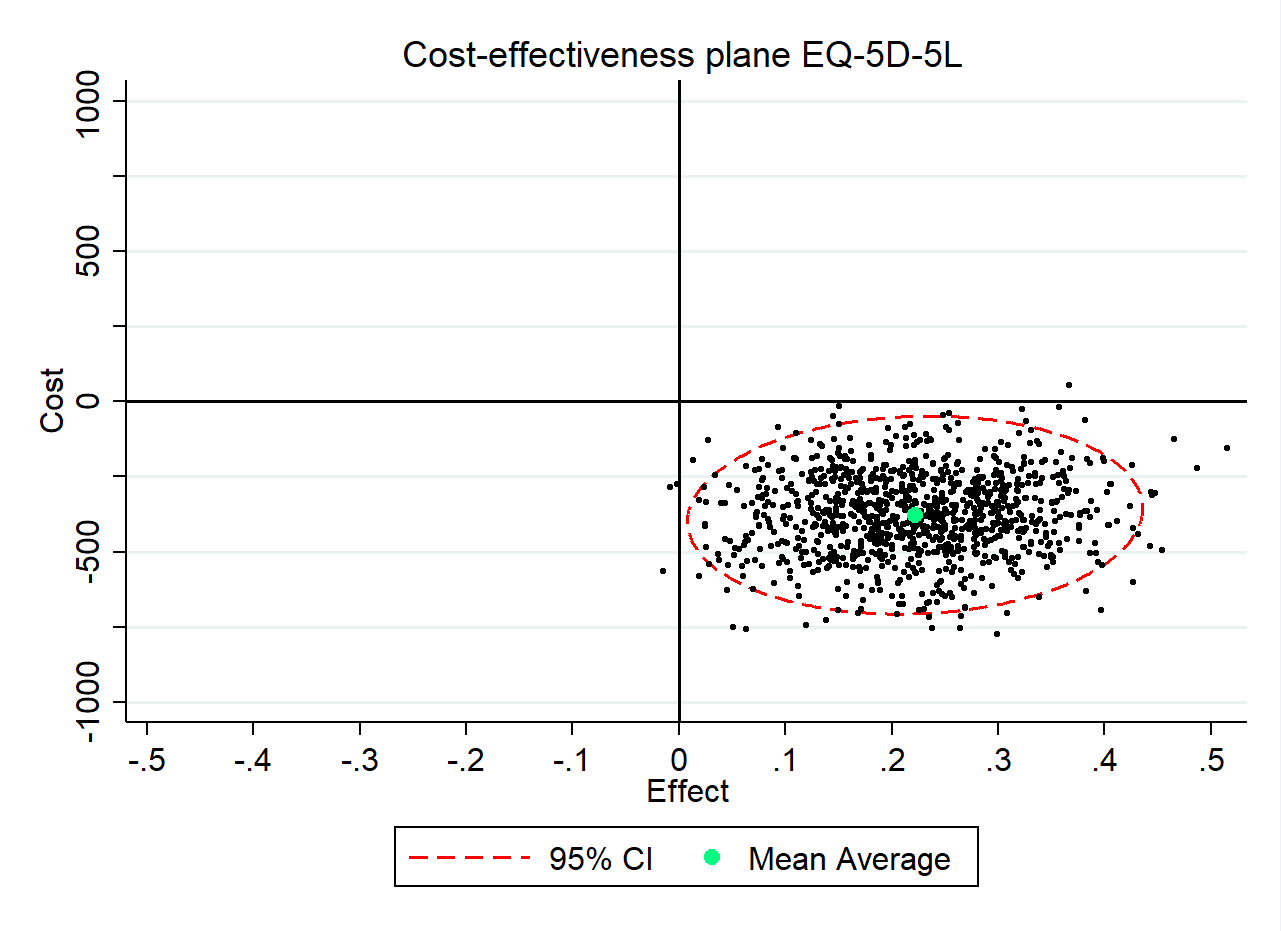

Supplement: S4 Fig — (TIF) [file pmed.1004459.s007.tif]

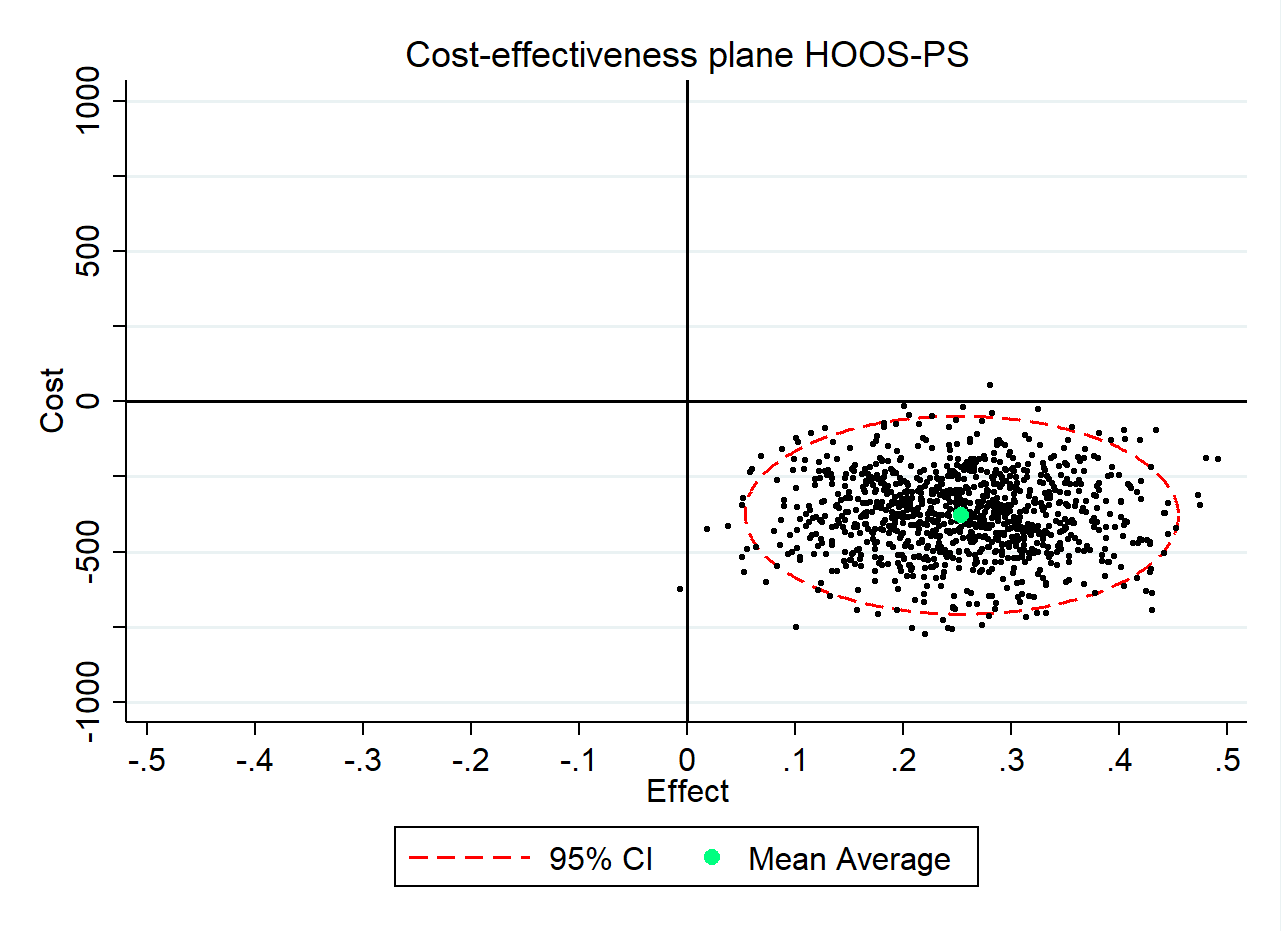

Supplement: S5 Fig — (TIF) [file pmed.1004459.s008.tif]

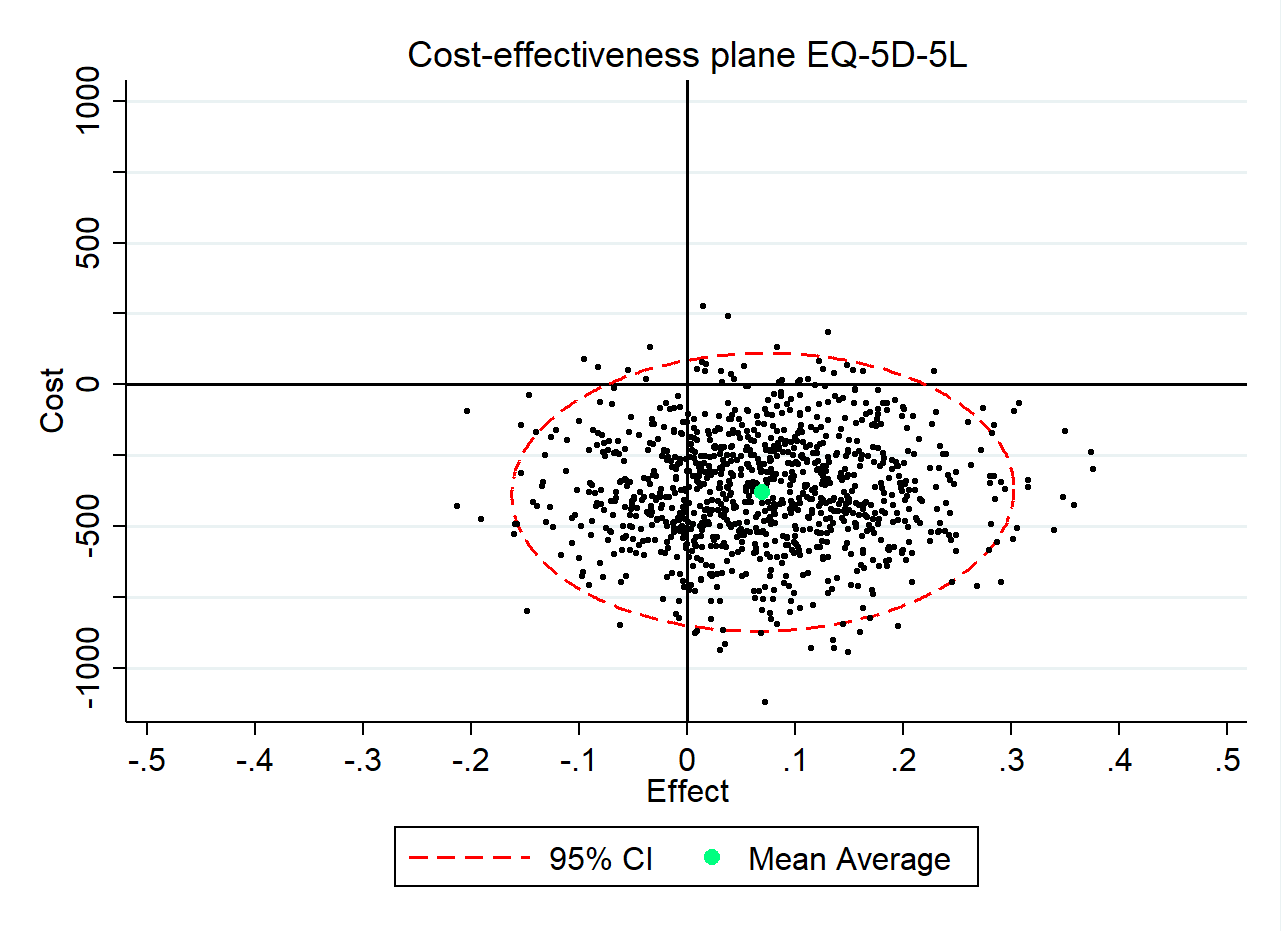

Supplement: S6 Fig — (TIF) [file pmed.1004459.s009.tif]

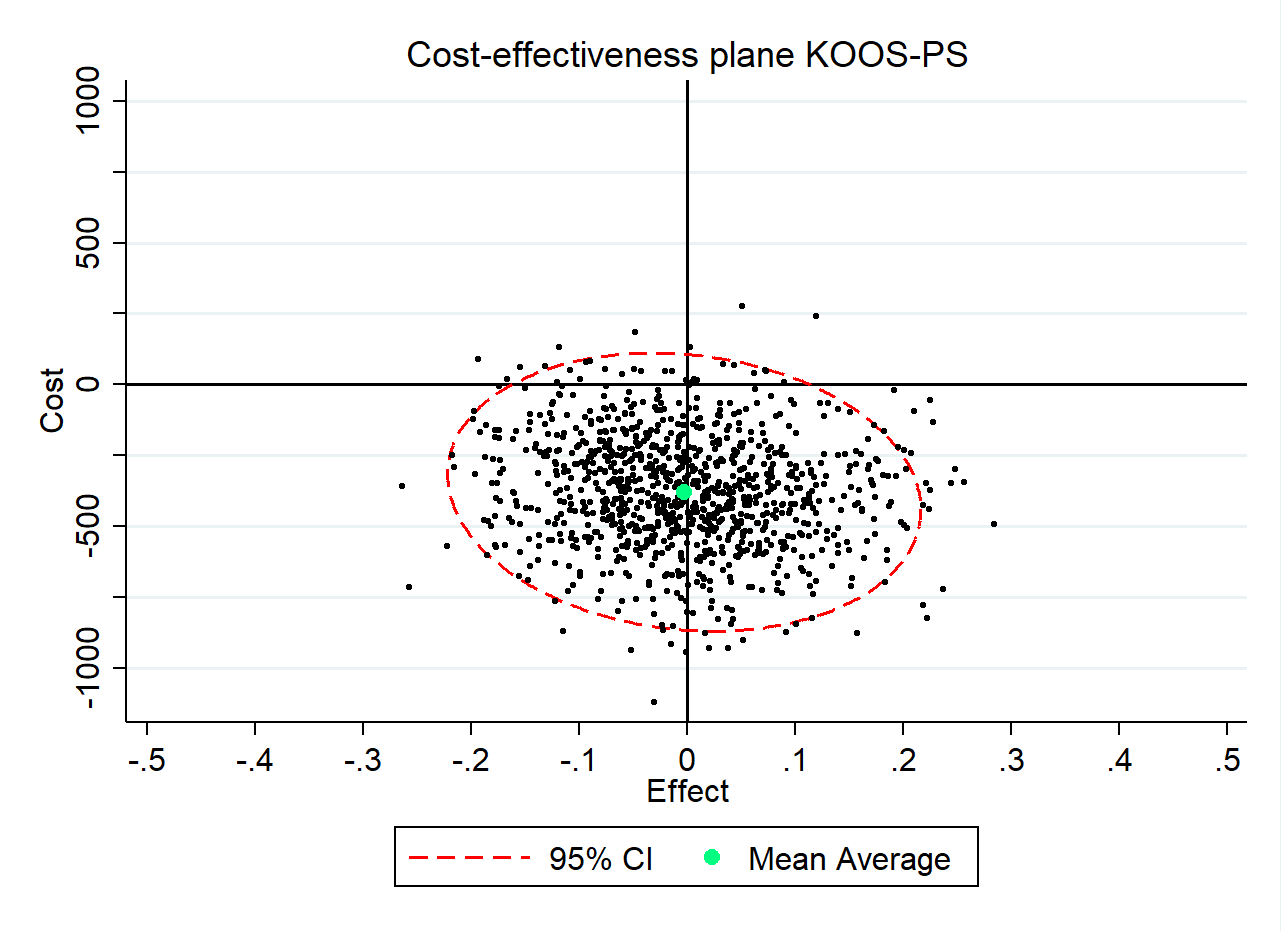

Supplement: S7 Fig — (TIF) [file pmed.1004459.s010.tif]
