# Supplementary material for: Cost-effectiveness of a patient-reported outcome-based remote monitoring and alert intervention for early detection of critical recovery after joint replacement: A randomised controlled trial
Source: PLoS Med. 2024 Oct 9;21(10):e1004459. doi: 10.1371/journal.pmed.1004459 (PMC11463742; doi:10.1371/journal.pmed.1004459)
Supplement: S1 Table — (DOCX) [file pmed.1004459.s011.docx]

| S1 Table – Procedure Codes (OPS) for inclusion of patients |
| --- |
| \| Procedure codes^a^ \| The following procedure codes were included:  Hip: 5-820.00, 5-820.01, 5-820.02, 5-820.20, 5-820.22, 5-820.8, 5-820.80, 5-820.81, 5-820.82, 5-820.9, 5-820.92, 5-820.93, 5-820.94, 5-820.95, 5-820.96, 5-820.X, 5-820.x0, 5-820.x1, 5-820.x2, 5-820.y  Knee: 5-822.0, 5-822.00, 5-822.01, 5-822.02, 5-822.g, 5-822.g0, 5-822.g1, 5-822.g2, 5-822.j, 5-822.j1, 5-822.j2, 5-822.k, 5-822.k0, 5-822.k1, 5-822.k2, 5-822.h1, 5-822.h2 \| \| --- \| --- \| |
| ^a^The German procedure classification (Operationen- und Prozedurenschlüssel - OPS) is the official classification for the encoding of operations, procedures and general medical measures. |
