# Supplementary material for: Cost-effectiveness of a patient-reported outcome-based remote monitoring and alert intervention for early detection of critical recovery after joint replacement: A randomised controlled trial
Source: PLoS Med. 2024 Oct 9;21(10):e1004459. doi: 10.1371/journal.pmed.1004459 (PMC11463742; doi:10.1371/journal.pmed.1004459)
Supplement: S2 Table — (DOCX) [file pmed.1004459.s012.docx]

| S2 Table – Absolute and relative intervention alert thresholds for each PROM-score |
| --- |
| \| **PROM Sets** \| **Month 1^a^** \| **Month 3^a^** \| **Month 6^a^** \| **Relative worsening alert^b^** \| **Relative Threshold** \| \| --- \| --- \| --- \| --- \| --- \| --- \| \| EQ-5D-5L hip \| 0.37 \| 0.64 \| 0.74 \| Yes \| 10% worse than t-1 \| \| HOOS-PS \| 53.00 \| 36.30 \| 27.70 \| Yes \| 10pt worse than t-1 \| \| EQ-5D-5L knee \| 0.36 \| 0.51 \| 0.70 \| Yes \| 10% worse than t-1 \| \| KOOS-PS \| 51.60 \| 43.60 \| 33.60 \| Yes \| 10pt worse than t-1 \| \| PROMIS-D-SF \| 65.80 \| 65.80 \| 65.80 \| No \| NA \| \| PROMIS-F-SF \| 69.00 \| 69.00 \| 69.00 \| No \| NA \| |
| PROM – Patient-Reported Outcome Measures; HOOS-PS – Hip Disability and Osteoarthritis Outcome Score Physical Function Short-form; KOOS-PS – Knee Injury and Osteoarthritis Outcome Score Physical Function Short-form; PROMIS – Patient-Reported Outcomes Measurement Information System Depression Shortform (PROMIS‐D‐SF) and Fatigue Shortform (PROMIS‐F‐SF)  ^a^ The absolute alert thresholds were set through a Delphi panel (Kuklinski et al. 2020)  ^b^ The relative change to a worse score initiated an alert for all PROM-scores with “Yes”. This table is based on the table published in the study protocol of the PROMoting Quality trial (Kuklinski et al. 2020) |
