# Supplementary material for: Cost-effectiveness of a patient-reported outcome-based remote monitoring and alert intervention for early detection of critical recovery after joint replacement: A randomised controlled trial
Source: PLoS Med. 2024 Oct 9;21(10):e1004459. doi: 10.1371/journal.pmed.1004459 (PMC11463742; doi:10.1371/journal.pmed.1004459)
Supplement: S3 Table — (DOCX) [file pmed.1004459.s013.docx]

| S3 Table - Critical values and alert reactions |
| --- |
| \|  \| **Hip replacement patients** \| \| \| **Knee replacement patients** \| \| \| \| --- \| --- \| --- \| --- \| --- \| --- \| --- \| \| month 1 \| month 3 \| month 6 \| month 1 \| month 3 \| month 6 \| \| **% of patients in the intervention group with information on thresholds** \| \| \| \| \| \| \| \| patients with critical values \| 9% \| 26% \| 25% \| 11% \| 26% \| 32% \| \| patients without critical values \| 91% \| 74% \| 75% \| 89% \| 74% \| 68% \| \| **number of those with critical values (% of critical value patients)** \| \| \| \| \| \| \| \| call to patient by study nurse \| 73% \| 70% \| 76% \| 71% \| 74% \| 69% \| \| data transfer to patients \| 18% \| 19% \| 20% \| 20% \| 19% \| 23% \| \| data transfer to physician \| 15% \| 9% \| 6% \| 18% \| 13% \| 5% \| \| physician visit \| 66% \| 66% \| 61% \| 65% \| 75% \| 70% \| |
|  |
