# Supplementary material for: Cost-effectiveness of a patient-reported outcome-based remote monitoring and alert intervention for early detection of critical recovery after joint replacement: A randomised controlled trial
Source: PLoS Med. 2024 Oct 9;21(10):e1004459. doi: 10.1371/journal.pmed.1004459 (PMC11463742; doi:10.1371/journal.pmed.1004459)
Supplement: S4 Table — (DOCX) [file pmed.1004459.s014.docx]

| S4 Table – Properties of the PRO measures |
| --- |
| \| **PROM** \| **Domain** \| **Range** \| **Directionality^a^** \| \| --- \| --- \| --- \| --- \| \| EQ-5D-5L \| HRQoL \| -0.661 – 1 \| Positive \| \| EQ-VAS \| HRQoL \| 0 – 100 \| Positive \| \| HOOS-PS \| Joint Functionality \| 0 – 100 \| Negative \| \| KOOS-PS \| Joint Functionality \| 0 – 100 \| Negative \| \| PROMIS-D-SF \| Mental Health \| 41.0 – 79.4 \| Negative \| \| PROMIS-F-SF \| Mental Health \| 33.7 – 75.8 \| Negative \| \| Analogue Pain Scales \| Pain Symptoms \| 0 – 10 \| Negative \| |
| PROM – Patient-Reported Outcome Measures; HOOS-PS – Hip Disability and Osteoarthritis Outcome Score Physical Function Short-form; KOOS-PS – Knee Injury and Osteoarthritis Outcome Score Physical Function Short-form; PROMIS – Patient-Reported Outcomes Measurement Information System Depression Shortform (PROMIS‐D‐SF) and Fatigue Shortform (PROMIS‐F‐SF); HRQoL – Healt-Related Quality of Life  ^a^ A positive directionality indicates that higher scores reflect better outcomes while with a negative directionality a higher score indicates worse symptoms, i.e. bad outcomes |
