# Supplementary material for: Cost-effectiveness of a patient-reported outcome-based remote monitoring and alert intervention for early detection of critical recovery after joint replacement: A randomised controlled trial
Source: PLoS Med. 2024 Oct 9;21(10):e1004459. doi: 10.1371/journal.pmed.1004459 (PMC11463742; doi:10.1371/journal.pmed.1004459)
Supplement: S5 Table — (DOCX) [file pmed.1004459.s015.docx]

| S5 Table – Calculation and pricing of personnel minutes |
| --- |
| \| \| Days per year  - weekends  - training days  - holidays  - national holidays (Berlin)  Gross working days per year \| 365  104  5  30  7  219 \| \| --- \| --- \| \| Hours per working day  - Time not on patients  Hours per year  - Absenteeism^1^  Net working hours per year \| 8  1  1533 (=219 x 7)  20 days = 120 h  1413 \| \| **Net working minutes per year** \| **84780** \| \| Mean gross income^2^ [€]  + EC health insurance^3^  + EC nursing care insurance^3^  + EC unemployment insurance^3^  + EC pension insurance^3^  + 13^th^ monthly salary  **Mean yearly income [€]** \| 39497  2883 (7.3 %)  592 (1.5 %)  474 (1.2 %)  3673 (9.3%)  3291  **50410** \| \| **Costs [€] per working minute** \| **0**.**59** \| \| \| --- \| --- \| --- \| --- \| --- \| --- \| --- \| --- \| --- \| --- \| --- \| \|  \| |
| EC – Employer contribution  ^1^ Source: AOK Fehlzeitenreport  ^2^ Source: Bundesagentur für Arbeit  ^3^ Source: Soialversicherungsbeiträge 2021 |
