# Supplementary material for: Cost-effectiveness of a patient-reported outcome-based remote monitoring and alert intervention for early detection of critical recovery after joint replacement: A randomised controlled trial
Source: PLoS Med. 2024 Oct 9;21(10):e1004459. doi: 10.1371/journal.pmed.1004459 (PMC11463742; doi:10.1371/journal.pmed.1004459)
Supplement: S6 Table — (DOCX) [file pmed.1004459.s016.docx]

| S6 Table – Calculation of the required staff minutes for the intervention |
| --- |
| \| **time** \| **occurence^a^** \| **tasks^b^** \| **minutes^c^** \| **incidence^d^** \| **sum^e^** \| \| --- \| --- \| --- \| --- \| --- \| --- \| \| Admission \| 1 time \| - Informative talk between medical staff and patients - Handing out token for sign-up \| 1 \| 100% \| 4 \| \| - Token sign-up - Baseline PROM questionnaire - Create patient file - Transfer patient data into hospital information system \| 3 \| \| Discharge \| 1 time \| - Automatically triggered follow-up PROM questionnaire per email \| 0 \| 100% \| 0 \| \| Follow-up \| 4 times \| - Automatically triggered follow-up PROM questionnaire per email - Personal reminder by medical staff in case of non-response \| 10 \| 20% \| 8 \| \| Intervention \| 3 times \| - Contacting patients in case of threshold exceedances - Transfer PROM results to patients \| 12 \| 24% \| 9 \| \| **Total time in min** \| \| \| \| \| **21** \| |
| ^a^how often tasks appear at the corresponding point of time  ^b^tasks for medical staff that arise in the corresponding time  ^c^number of minutes for the tasks; number of minutes were derived from structured interviews with the study nurses of the PROMoting Quality trial  ^d^approx. share of affected patients; number derived from the PROMoting Quality sample  ^e^calculated as occurrence x minutes x incidence |
