# Supplementary material for: Cost-effectiveness of a patient-reported outcome-based remote monitoring and alert intervention for early detection of critical recovery after joint replacement: A randomised controlled trial
Source: PLoS Med. 2024 Oct 9;21(10):e1004459. doi: 10.1371/journal.pmed.1004459 (PMC11463742; doi:10.1371/journal.pmed.1004459)
Supplement: S7 Table — (DOCX) [file pmed.1004459.s017.docx]

| S7 Table – Post-surgery costs for hip replacement patients |
| --- |
| \|  \| \| Intervention (n=284) \| \| \| \| Control (n=262) \| \| \| \| Comparative statistic^a^ \| \| \| --- \| --- \| --- \| --- \| --- \| --- \| --- \| --- \| --- \| --- \| --- \| --- \| \| mean \| SD \| median \| IQR \| mean \| SD \| median \| IQR \| p (t) \| p (w) \| \| OC \| Utilisation n (%) ^b^ \| 284 (100.00%) \| \| \| \| 261 (99.62%) \| \| \| \| 0.298 \| 0.298 \| \| Cases ^c^ \| 11.91 \| 5.96 \| 11 \| 7 \| 13.51 \| 6.83 \| 13 \| 9 \| 0.004 \| 0.007 \| \| Raw in € ^d^ \| 852.27 \| 856.52 \| 658.70 \| 626.52 \| 1026.72 \| 1075.65 \| 807.92 \| 767.95 \| 0.036 \| 0.003 \| \| Adjusted in € ^e^ \| 816.43 \| 329.89 \| 740.13 \| 422.10 \| 907.01 \| 360.41 \| 817.18 \| 539.86 \| 0.002 \| 0.002 \| \| OHC \| Utilisation n (%) ^b^ \| 21 (7.39%) \| \| \| \| 21 (8.02%) \| \| \| \| 0.786 \| 0.786 \| \| Cases ^c^ \| 0.15 \| 0.63 \| 0 \| 0 \| 0.15 \| 0.61 \| 0 \| 0 \| 0.853 \| 0.823 \| \| Raw in € ^d^ \| 32.00 \| 145.72 \| 0.00 \| 0.00 \| 40.29 \| 217.47 \| 0 \| 0 \| 0.598 \| 0.784 \| \| Adjusted in € ^e^ \| 13.85 \| 6.58 \| 11.60 \| 5.43 \| 14.67 \| 7.47 \| 12.39 \| 6.19 \| 0.174 \| 0.240 \| \| IC \| Utilisation n (%) ^b^ \| 74 (26.06%) \| \| \| \| 77 (29.39%) \| \| \| \| 0.385 \| 0.385 \| \| Cases ^c^ \| 0.36 \| 0.71 \| 0 \| 1 \| 0.46 \| 0.84 \| 0 \| 1 \| 0.122 \| 0.261 \| \| Raw in € ^d^ \| 1735.18 \| 4404.33 \| 0.00 \| 437.48 \| 2055.79 \| 4548.14 \| 0.00 \| 211.16 \| 0.403 \| 0.352 \| \| Adjusted in € ^e^ \| 1438.52 \| 440.01 \| 1332.25 \| 541.30 \| 1551.83 \| 476.87 \| 1403.10 \| 714.14 \| 0.004 \| 0.003 \| \| PRES \| Utilisation n (%) ^b^ \| 272 (95.77%) \| \| \| \| 250 (95.42%) \| \| \| \| 0.840 \| 0.840 \| \| Cases ^c^ \| 14.05 \| 12.69 \| 11 \| 14 \| 16.52 \| 14.20 \| 14 \| 16 \| 0.032 \| 0.022 \| \| Raw in € ^d^ \| 745.82 \| 1775.37 \| 280.56 \| 649.58 \| 1223.32 \| 5054.07 \| 364.97 \| 690.06 \| 0.135 \| 0.031 \| \| Adjusted in € ^e^ \| 560.32 \| 586.21 \| 331.51 \| 444.96 \| 654.50 \| 641.81 \| 404.54 \| 570.68 \| 0.074 \| 0.023 \| \| REM \| Utilisation n (%) ^b^ \| 239 (84.15%) \| \| \| \| 227 (86.64%) \| \| \| \| 0.413 \| 0.412 \| \| Cases ^c^ \| 8.36 \| 8.58 \| 6 \| 8 \| 10.09 \| 10.01 \| 8 \| 11 \| 0.031 \| 0.041 \| \| Raw in € ^d^ \| 689.03 \| 903.64 \| 463.89 \| 652.65 \| 867.40 \| 1217.52 \| 563.37 \| 843.16 \| 0.051 \| 0.019 \| \| Adjusted in € ^e^ \| 658.19 \| 294.63 \| 560.95 \| 334.55 \| 722.09 \| 318.29 \| 622.61 \| 437.48 \| 0.015 \| 0.010 \| \| AIDS \| Utilisation n (%) ^b^ \| 175 (61.62%) \| \| \| \| 172 (65.65%) \| \| \| \| 0.329 \| 0.329 \| \| Cases ^c^ \| 2.36 \| 3.64 \| 1 \| 3 \| 2.66 \| 4.01 \| 1 \| 3 \| 0.352 \| 0.388 \| \| Raw in € ^d^ \| 171.96 \| 408.35 \| 56.31 \| 173.90 \| 218.69 \| 661.61 \| 61.19 \| 200.77 \| 0.317 \| 0.320 \| \| Adjusted in € ^e^ \| 132.82 \| 64.35 \| 112.75 \| 63.71 \| 146.47 \| 67.95 \| 124.89 \| 80.65 \| 0.016 \| 0.005 \| \| **Total** \| Raw in € ^d^ \| **4226.26** \| **5575.47** \| **2146.55** \| **3946.5** \| **5432.22** \| **7604.43** \| **2614.23** \| **5918.04** \| **0.034** \| **0.019** \| \| Adjusted in € ^e^ \| **3620.12** \| **1544.80** \| **3218.23** \| **1619.8** \| **3996.55** \| **1656.16** \| **3508.19** \| **2312.27** \| **0.006** \| **0.004** \| |
| OC – Outpatient care; OHC – Outpatient hospital care; IC – Inpatient care; PRES – Prescriptions; REM – Remedies; AIDS – medical aids;  ^a^Comparative Analysis was conducted at 5% level with two-sided t-tests (p(t)) and, in case of non-normality, with wilcoxon rank-sum tests (p(w))  ^b^if a service in the corresponding category was used at least once in the 1-year post-surgery period  ^c^number of cases per category in the 1-year post-surgery period  ^d^unadjusted occurred costs the 1-year post-surgery period  ^e^1-year post-surgery period costs adjusted for the baseline differences with winsorised linear regression |
