# Supplementary material for: Cost-effectiveness of a patient-reported outcome-based remote monitoring and alert intervention for early detection of critical recovery after joint replacement: A randomised controlled trial
Source: PLoS Med. 2024 Oct 9;21(10):e1004459. doi: 10.1371/journal.pmed.1004459 (PMC11463742; doi:10.1371/journal.pmed.1004459)
Supplement: S8 Table — (DOCX) [file pmed.1004459.s018.docx]

| S8 Table – Post-surgery costs for knee replacement patients |
| --- |
| \|  \| \| intervention (n=284) \| \| \| \| control (n=262) \| \| \| \| Comparative statistic^a^ \| \| \| --- \| --- \| --- \| --- \| --- \| --- \| --- \| --- \| --- \| --- \| --- \| --- \| \| mean \| SD \| median \| IQR \| mean \| SD \| median \| IQR \| p (t) \| p (w) \| \| OC \| Utilisation n (%) ^b^ \| 236 (99.16%) \| \| \| \| 254 (100%) \| \| \| \| 0.144 \| 0.144 \| \| Cases ^c^ \| 14.33 \| 7.23 \| 13.00 \| 10.00 \| 15.16 \| 7.47 \| 14.00 \| 9.00 \| 0.210 \| 0.225 \| \| Raw in € ^d^ \| 1027.76 \| 854.72 \| 743.31 \| 848.29 \| 1121.89 \| 1263.25 \| 860.37 \| 839.35 \| 0.337 \| 0.329 \| \| Adjusted in € ^e^ \| 965.15 \| 411.98 \| 831.82 \| 526.71 \| 1034.26 \| 418.81 \| 949.94 \| 540.56 \| 0.066 \| 0.028 \| \| OHC \| Utilisation n (%) ^b^ \| 22 (9.24%) \| \| \| \| 36 (14.17%) \| \| \| \| 0.091 \| 0.091 \| \| Cases ^c^ \| 0.12 \| 0.41 \| 0.00 \| 0.00 \| 0.25 \| 0.88 \| 0.00 \| 0.00 \| 0.032 \| 0.076 \| \| Raw in € ^d^ \| 26.39 \| 142.94 \| 0.00 \| 0.00 \| 48.74 \| 168.50 \| 0.00 \| 0.00 \| 0.114 \| 0.074 \| \| Adjusted in € ^e^ \| 20.72 \| 15.24 \| 15.29 \| 17.02 \| 23.01 \| 17.56 \| 15.36 \| 21.65 \| 0.124 \| 0.315 \| \| IC \| Utilisation n (%) ^b^ \| 76 (31.93%) \| \| \| \| 90 (35.43%) \| \| \| \| 0.413 \| 0.415 \| \| Cases ^c^ \| 0.47 \| 0.78 \| 0.00 \| 1.00 \| 0.55 \| 0.91 \| 0.00 \| 1.00 \| 0.294 \| 0.412 \| \| Raw in € ^d^ \| 1948.25 \| 4438.04 \| 0.00 \| 253.09 \| 2716.69 \| 6867.13 \| 0.00 \| 2954.12 \| 0.144 \| 0.304 \| \| Adjusted in € ^e^ \| 1890.02 \| 701.86 \| 1669.25 \| 847.94 \| 2011.77 \| 707.99 \| 1854.54 \| 979.75 \| 0.056 \| 0.020 \| \| PRES \| Utilisation n (%) ^b^ \| 233 (97.90%) \| \| \| \| 251(98.82%) \| \| \| \| 0.421 \| 0.421 \| \| Cases ^c^ \| 18.49 \| 14.95 \| 15.00 \| 16.00 \| 21.88 \| 16.52 \| 19.00 \| 18.00 \| 0.018 \| 0.010 \| \| Raw in € ^d^ \| 997.81 \| 2152.47 \| 374.85 \| 724.90 \| 1261.99 \| 2830.65 \| 535.74 \| 820.94 \| 0.247 \| 0.017 \| \| Adjusted in € ^e^ \| 785.98 \| 791.37 \| 473.29 \| 611.31 \| 907.93 \| 845.99 \| 560.35 \| 810.62 \| 0.100 \| 0.038 \| \| REM \| Utilisation n (%) ^b^ \| 215 (90.34%) \| \| \| \| 233 (91.73%) \| \| \| \| 0.589 \| 0.588 \| \| Cases ^c^ \| 11.41 \| 9.85 \| 9.00 \| 12.00 \| 13.12 \| 12.23 \| 10.00 \| 13.00 \| 0.089 \| 0.272 \| \| Raw in € ^d^ \| 1019.19 \| 1005.56 \| 708.38 \| 1033.68 \| 1195.83 \| 1412.72 \| 769.65 \| 1066.68 \| 0.113 \| 0.301 \| \| Adjusted in € ^e^ \| 229.28 \| 147.03 \| 178.97 \| 161.95 \| 253.05 \| 158.45 \| 199.81 \| 217.43 \| 0.086 \| 0.093 \| \| AIDS \| Utilisation n (%) ^b^ \| 135 (56.72%) \| \| \| \| 162 (63.78%) \| \| \| \| 0.110 \| 0.110 \| \| Cases ^c^ \| 2.22 \| 3.61 \| 1.00 \| 3.00 \| 3.08 \| 5.08 \| 1.00 \| 4.00 \| 0.032 \| 0.031 \| \| Raw in € ^d^ \| 278.80 \| 808.19 \| 37.83 \| 225.58 \| 335.27 \| 881.04 \| 81.00 \| 323.53 \| 0.460 \| 0.047 \| \| Adjusted in € ^e^ \| 1016.48 \| 473.84 \| 859.83 \| 466.46 \| 1053.11 \| 521.89 \| 858.09 \| 492.42 \| 0.416 \| 0.760 \| \| **Total** \| Raw in € ^d^ \| **5298.20** \| **6041.66** \| **3179.92** \| **4592.80** \| **6680.41** \| **8645.18** \| **3555.63** \| **7387.66** \| **0.042** \| **0.074** \| \| Adjusted in € ^e^ \| **4907.62** \| **2186.07** \| **4221.53** \| **2409.09** \| **5283.13** \| **2271.83** \| **4633.52** \| **2961.50** \| **0.063** \| **0.034** \| |
| OC – Outpatient care; OHC – Outpatient hospital care; IC – Inpatient care; PRES – Prescriptions; REM – Remedies; AIDS – medical aids;  ^a^Comparative Analysis was conducted at 5% level with two-sided t-tests (p(t)) and, in case of non-normality, with wilcoxon rank-sum tests (p(w))  ^b^if a service in the corresponding category was used at least once in the 1-year post-surgery period  ^c^number of cases per category in the 1-year post-surgery period  ^d^unadjusted occurred costs the 1-year post-surgery period  ^e^1-year post-surgery period costs adjusted for the baseline differences with winsorised linear regression |
