# Supplementary material for: Cost-effectiveness of a patient-reported outcome-based remote monitoring and alert intervention for early detection of critical recovery after joint replacement: A randomised controlled trial
Source: PLoS Med. 2024 Oct 9;21(10):e1004459. doi: 10.1371/journal.pmed.1004459 (PMC11463742; doi:10.1371/journal.pmed.1004459)
Supplement: S9 Table — (DOCX) [file pmed.1004459.s019.docx]

| S9 Table – Descriptive Statistics of the whole study sample |
| --- |
| \|  \| **Hip replacement patients** \| \| **Knee replacement patients** \| \| \| --- \| --- \| --- \| --- \| --- \| \| **intervention (N=1,854)** \| **control (N=1,843)** \| **intervention**  **(N=1,564)** \| **control**  **(N=1,546)** \| \| **age** \| \| \| \| \| \| mean (SD) \| 65.9 (10.6) \| 65.7 (10.6) \| 66.1 (9.09) \| 65.9 (9.36) \| \| **gender (%)** \| \| \| \| \| \| female \| 1,029 (55.5) \| 1,036 (56.2) \| 839 (53.6) \| 830 (53.7) \| \| male \| 825 (44.5) \| 807 (43.8) \| 725 (46.4) \| 716 (46.3) \| \| **bmi group (%)** \| \| \| \| \| \| underweight \| 10 (0.5) \| 10 (0.5) \| 3 (0.2) \| 3 (0.2) \| \| normal \| 576 (31.1) \| 588 (31.9) \| 255 (16.3) \| 222 (14.4) \| \| overweight \| 722 (38.9) \| 699 (37.9) \| 566 (36.2) \| 618 (40.0) \| \| obese \| 546 (29.4) \| 546 (29.6) \| 740 (47.3) \| 703 (45.5) \| \| **current smoker (%)** \| \| \| \| \| \| no \| 1,586 (85.5) \| 1,547 (83.9) \| 1,369 (87.5) \| 1,336 (86.4) \| \| yes \| 268 (14.5) \| 296 (16.1) \| 195 (12.5) \| 210 (13.6) \| \| **education (%)** \| \| \| \| \| \| no school degree \| 8 (0.4) \| 7 (0.4) \| 13 (0.8) \| 5 (0.3) \| \| primary school degree \| 243 (13.1) \| 249 (13.5) \| 263 (16.8) \| 271 (17.5) \| \| high/middle school degree \| 1,085 (58.5) \| 1,033 (56.1) \| 932 (59.6) \| 927 (60.0) \| \| university degree \| 518 (27.9) \| 554 (30.1) \| 356 (22.8) \| 343 (22.2) \| \| **living situation (%)** \| \| \| \| \| \| alone \| 430 (23.2) \| 414 (22.5) \| 310 (19.8) \| 289 (18.7) \| \| care facility \| 4 (0.2) \| 7 (0.4) \| 10 (0.6) \| 9 (0.6) \| \| with a partner/family/friends \| 1,411 (76.1) \| 1,408 (76.4) \| 1,239 (79.2) \| 1,233 (79.8) \| \| other \| 9 (0.5) \| 14 (0.8) \| 5 (0.3) \| 15 (1.0) \| \| **job (%)** \| \| \| \| \| \| working \| 583 (31.4) \| 618 (33.5) \| 454 (29) \| 468 (30.3) \| \| voluntarily not working including retirement \| 1,065 (57.4) \| 1,034 (56.1) \| 915 (58.5) \| 875 (56.6) \| \| looking for work \| 20 (1.1) \| 15 (0.8) \| 11 (0.7) \| 23 (1.5) \| \| unable to work \| 186 (10) \| 176 (9.6) \| 184 (11.7) \| 180 (11.6) \| \| **mobilization after surgery (%)** \| \| \| \| \| \| within 6 hours \| 854 (46.1) \| 854 (46.3) \| 716 (45.8) \| 709 (45.9) \| \| within 12 hours \| 560 (30.2) \| 496 (26.9) \| 460 (29.4) \| 425 (27.5) \| \| within 24 hours \| 403 (21.7) \| 443 (24.0) \| 331 (21.2) \| 356 (23.0) \| \| within 48 hours \| 26 (1.4) \| 35 (1.9) \| 43 (2.7) \| 46 (3.0) \| \| after 48 hours \| 11 (0.6) \| 15 (0.8) \| 14 (0.9) \| 10 (0.6) \| \| **readmission (%)** \| \| \| \| \| \| no \| 1,809 (97.6) \| 1,789 (97.1) \| 1,531 (97.9) \| 1,506 (97.4) \| \| within 30 days post-surgery \| 19 (1.0) \| 28 (1.5) \| 9 (0.6) \| 21 (1.4) \| \| 30-90 days post-surgery \| 26 (1.4) \| 26 (1.4) \| 24 (1.5) \| 19 (1.2) \| \| **reoperation within 12 months post-surgery (%)** \| \| \| \| \| \| no \| 1,816 (98.0) \| 1,798 (97.6) \| 1,530 (97.8) \| 1,504 (97.3) \| \| yes \| 38 (2.0) \| 45 (2.4) \| 34 (2.2) \| 42 (2.7) \| \| **PROM baseline score means (SD)^a^** \| \| \| \| \| \| EQ-5D-5L \| 0.594 (0.263) \| 0.603 (0.256) \| 0.625 (0.257) \| 0.623 (0.245) \| \| EQ-VAS \| 56.5 (20.0) \| 57.1 (19.7) \| 58.7 (19.4) \| 57.9 (19.1) \| \| HOOS/KOOS-PS \| 48.4 (16.4) \| 47.1 (16.1) \| 43.1 (13.3) \| 43.0 (12.0) \| \| PROMIS-fatigue \| 49.2 (9.91) \| 49.2 (9.98) \| 48.3 (10.1) \| 48.1 (9.54) \| \| PROMIS-depression \| 49.7 (8.35) \| 49.8 (8.26) \| 49.4 (8.39) \| 49.4 (8.16) \| |
| *BMI – Body Mass Index; PROM – Patient-Reported Outcome Measures; SD – Standard Deviation; EQ-5D-5L – EuroQol five dimensions, five levels; EQ-VAS – EuroQol virtual analogue scale; HOOS-PS – Hip Disability and Osteoarthritis Outcome Score Physical Function Short-form; KOOS-PS – Knee Injury and Osteoarthritis Outcome Score Physical Function Short-form; PROMIS – Patient-Reported Outcomes Measurement Information System Depression Shortform (PROMIS‐D‐SF) and Fatigue Shortform (PROMIS‐F‐SF)* |
