# Supplementary material for: Cost-effectiveness of a patient-reported outcome-based remote monitoring and alert intervention for early detection of critical recovery after joint replacement: A randomised controlled trial
Source: PLoS Med. 2024 Oct 9;21(10):e1004459. doi: 10.1371/journal.pmed.1004459 (PMC11463742; doi:10.1371/journal.pmed.1004459)
Supplement: Attachment — Submitted filename: 20240422_revisions_response.docx [file pmed.1004459.s020.docx]

Dear editor, dear reviewers,

Thank you very much again for your valuable comments. They have helped us to substantially improve the article. We hope to have included your feedback to your satisfaction. In this document we respond to each of the reviewers´ feedback. Below the reviewers´ comments, we provide our response in blue and describe how we chose to address the feedback. Line references in the responses refer to the marked-up copy of the manuscript.

Please let us know if you have additional feedback or comments. Thank you again for your help in making this a better research contribution.

Yours sincerely,

Lukas Schöner

**Editorial comments**

The premise and idea are strong, and a long-term evaluation of arthroplasty using PROMS is a reasonable approach. The background and setting are appropriate and reasonable, the methodology appears correct (in the main), and it is good to see an efficient, partially embedded, registry-style trial. The study is generally rigorous, there is good attention to detail, and the paper is well written and organized.

***Author response:*** *Thank you very much for this positive feedback. We are very pleased that our research has aroused your interest and appreciate your thorough review very much.*

There are 3 slightly larger concerns:

1) The sample size is good, but there is no a priori power for the expected effect - this is always required for a prospective RCT, even if it is partially embedded.

***Author response:*** *Many thanks for this comment. We realize that the details of the preceding power calculation can easily be overlooked. In the body text of the manuscript, we refer to the statistical analysis plan, which can be found in S1 Appendix. The a priori power calculation is listed there in detail. In order to improve the reading flow of the main manuscript, we have decided to move the details to the appendix. Nevertheless, in the following we would like to go into more detail about the a priori power calculation and the set effect size:*

- *Effect size: The effect size was set based on rather conservative assumptions as, firstly, valid estimations for an intervention effect were not possible in advance due to a lack of similar studies with a PROM composite measure and cost incurred for hip and knee replacement patients. Secondly, we assumed the effect from the PROM-based monitoring and alert system to be relatively low against the background of the comparatively limited intervention at patient level – in the intervention group within the one-year follow-up period, only three more PROM surveys were scheduled than in the control group. Further actions then only took place depending on (abnormal) results of the PROM survey. Therefore we assumed an intervention effect size of 0.15 standard deviations.*
- *Power of intervention: We assumed a priori a sufficiently high probability (power of 80%) for the statistical validity of an intervention effect with the common error probability of p = 5%. Assuming this effect size and power of the intervention on the "outcome over cost ratio", the minimum number of study participants required was determined to be n = 1396.*

*We hope that we could sufficiently illustrate our a priori power calculation with this description. To also enhance the comprehensibility to the reader, we have integrated a supplementary comment into the text in line 220-221 referring to the power calculation in the appendix. However, if you think the provided information in the manuscript does not suffice to provide the needed information to the reader, we are happy to further include a brief description of the power calculation in the main text.*

2) The study is driven from a Health Economics perspective. This has advantages, but also some limitations. The main problem here is that the sample (representative for the study) consists only of those patients with HE data. Since the HE data group is in the minority for the entire registry population, this significantly limits the external validity for any broader interpretation. Therefore, we do not know if the HE data group has different characteristics than the others. This does not make the paper useless by any means (the information and findings are still very valuable - and there is no evidence of differences), but it is a strong caveat to any authoritative conclusion. Please expand or discuss this point to a greater degree and maybe base their conclusions with clear reference to (a population with HE data available only).

***Author response:*** *Thank you very much, we really appreciate this advice. In the original version, we only briefly mention "different sample characteristics". In the revised version we provide more detail and added some helpful additional information that describe the differences to the overall study population. The main difference between our sample and the overall sample lies in the gender distribution. There is a higher proportion of female patients in our sample. This could at least partially explain the differences in the effectiveness of the intervention compared to the overall sample. As we have already highlighted in our study Langenberger et al. [1], particularly female patients benefit from the intervention. We further included the recent publication of Steinbeck et al. [2] who also found that the focus on gender-specific differences needs to be sharpened in the future. In this respect, we can partially explain the differences between this sample and the entire sample, but apart from this we only see minor differences in the sample characteristics, which generally indicates a high external validity of the results.
We discuss this matter in detail from line 393-405 and point out more information on the differences between this and the whole sample in line 398-405. Also, we refer more clearly to our published secondary analysis of the PROMoting Quality trial, where the effectiveness was tested for the overall sample (without costs). Lastly, to transparently disclose the differences, we also included an additional table (S9 Table) in the supplemental material that shows the descriptive statistics of the overall study population. We refer to this table in line 398.*

3) The restriction and emphasis on HE is also a problem for a wider perspective. It is important to know whether the system is good at picking up events, problems or SAEs. There is no data on this, only the line;

o [258] “Within 1-year post-surgery adverse events ratios (readmission and reoperation) were generally low and [259] showed no significant differences between control and intervention groups for both procedures.

It would therefore be good to see the values and comparative data for this statement in the results section. It is a critical aspect of the proposed system.

***Author response:*** *Thank you for this comment. Originally, we expected to see an intervention effect on adverse events, such as reoperations or readmissions as it enables a timely reaction to critical recovery paths or avoidance of “false alarms”. However, we found that the numbers of adverse events were generally very low in the whole sample as well as in our health economic sample. As the observed number of adverse events was so low, we decided to not include the exact number in the text, but only to refer to the values in the table. Nevertheless, although not statistically significant, a tendency in favor of the intervention group can be seen in both hip and knee patients. In this respect, and following your suggesting, we are now including the exact data in the manuscript in order to better substantiate the statement you mentioned. This tendency can also be seen in roughly equal proportions in the entire sample - the percentages remain similar. Our corresponding adjustments in the manuscript can be found in line 323-328.*

**Reviewer #1**

1) Data cannot be publicly disclosed due to restrictions, and the authors CP and FC's affiliations with private medical device and solution companies warrant further examination.

***Author response:*** *We are afraid that this is true, data cannot be made publicly available due to German data protection law and the data protection agreements within the trial. However, the data will be stored on a server that can be accessed under special requirements to enable verifiability of the results.*

*Further, we are aware that the affiliations of CP and FC with private medical device companies might reflect possible conflict of interests. However, both authors in no way were involved in the conceptualization of the manuscript and had no influence on the data analysis, methodology or results. As PROMoting Quality project partners, however, they both played an important role for the project management and data acquisition, and with this contributed an essential part for the success of the clinical trial.*

*CP is affiliated with a medical device company outside the submitted work. With regard to this research article, CP acted within his role as researcher at the Technical University of Berlin. His role in the medical device company did not influence the study design nor the results and implications.*

*FC is affiliated with the study’s software solution provider heartbeat medical. Within his role he was responsible for the data collection at the clinical partners and the technical project management; each step followed the predefined study protocol. However, he was not involved in the conceptualization of the study, nor the data analyses and implication of results.*

*All their contributions to this manuscript were done outside their affiliations and they did not receive any funds or grants that are in context with this manuscript. Therefore, they sufficiently meet the ICMJE requirements for co-authorship (but neither for lead authorship nor two senior authorship) and we can confidently exclude that their affiliations have in any way influenced the outcome of the study.*

2) Title: It might be better to avoid using acronyms (PROM) in the title.

***Author response:*** *Thank you for that advise. We changed the title accordingly to “Cost-effectiveness of a patient-reported outcome based remote monitoring and alert intervention for early detection of critical recovery after joint replacement: A randomised controlled trial” avoiding the use of the acronym PROM. We decided to use “patient-reported outcome” rather than “patient-reported outcome measure” as the intervention is rather based on the outcome (i.e. the change of PROM scores between two measurement points) than on the measure (i.e. the questionnaire per se) only.*

3) Abstract: It would be better to provide the full name of PROM when it first appears in the abstract.

**Author response:** Thank you. We changed it to the full term “Patient-reported outcome measure” the first time it appears in the abstract in line 35.

4) Line 68-70, introduction: The numbers are given, but there is little discussion of their implications or how they compare to other regions or time periods. It would be helpful to provide a brief comparison of these statistics to give the reader a sense of how they compare to other countries or regions.

***Author response:*** *Thank you for the advice. We intended to make the link to the German healthcare system as early as possible in the text, as the study took place in the German context. However, we agree that it is difficult to put these numbers into context without comparative data. We have therefore also included the OECD average numbers in order to generate a better understanding. The revised section can be found in line 104-107.*

5) Line 80-81, introduction: the authors have identified the gap in the literature but could further explain how this gap impacts clinical practice and policy decisions to underscore the importance of the research.

***Author response:*** *Thank you. We integrated further explanations and implications to the text to provide more potential levels of a PROM based intervention in orthopedics. The adjustments are made in line 119-124.*

6) Line 95-96, Method: What do ASA categories 4-6 signify? Are those severe cases? Providing detailed explanations of these categories would help readers better understand the rationale for the exclusion criteria.

***Author response:*** *Thank you, we agree that more details can enhance the comprehensibility. In order to give the reader a better understanding of what the ASA classifications 4-6 mean, we included a more detailed description of these in parentheses. Additionally, we included a reference from the American Society of Anesthesiologists Classification to give further detailed background information on this classification. Find the adjustments in the section Study design and participants in line 144-146.*

7) Line 96-97, Method: I am curious what is the proportion of patients who lack an email account or the ability to use digital PROMs? Given that patients undergoing HR or KR are mostly elderly individuals, who may not be adept with modern technology, there are concerns regarding the representativeness of the patient sample included in this study.

***Author response:*** *Thank you very much, a very interesting question. Unfortunately, we are unable to provide the exact numbers on that as we exclusively obtained data for included patients. Even though your assumption seems reasonable that older patients in particular are not used to using digital applications, there was also the option of getting assistance from relatives. As a result, it was also possible to include the patients in the study who did not have their own computer or email account. However, we assume that with an average age of 66, most patients had sufficient knowledge to be able to use the application or received help from relatives. Therefore, we assume a low exclusion rate due to lack of computer access, lack of email account or insufficient digital literacy. In future patient populations, it can also be assumed that digital literacy will increase and problems in this regard will decrease. In addition, the (digital) effort for the participants was kept very low and only a low level of digital literacy was required. The PROM questionnaires were sent by email, could be completed easily and intuitively within 10 minutes and did not require any special software.*

8) Line 106-107, Method: It would be beneficial to include a description of Heartbeat ONE software. Clarifying what this software is and its functions would help readers understand how it is used within the context of the study.

***Author response:*** *Thank you, we agree that it is beneficial to include more background information about the functioning of the heartbeat software. We therefore have included a brief description of the software in S2 Appendix in the supplementary material. The reference to this description can be found in line 158-159.*

9) Line 113-126, Method: it is mentioned that patients in the intervention group received additional follow-ups at 1, 3, and 6 months. There is a concern that these extra check-ins could influence the PROMs due to a potential perception among patients of receiving increased attention and care.

***Author response:*** *Thank you for pointing that out. We will gladly go into more detail here. We consider these additional follow-ups in the intervention group to be the actual core of the intervention. Thereby we expect these additional PROM follow-ups to have positive health effects through two different channels. If in the post-surgery period critical recovery paths were detected, further action took place, first through calls from the study nurses and second, if deemed necessary, through subsequent potential treatment adjustments. However, our hypothesis is that not only such an early reaction to changes in health status alone can have a positive effect on PROMs. Rather, we assume, and conclude from the results, that only answering PROMs, even without subsequent treatment adjustments, already has a positive effect, purely because patients feel more comfortable. In this context, we are referring to a "being taken care of" effect that PROMs can achieve simply by responding them (see line 394). If the mere perception of increased attention and care alone already has positive effects on physical and mental well-being, this represents an important result for this study and the effectiveness of PROMs in clinical practice. However, we hope that the description of the procedures is generally understandable - for a better understanding for the reader, we have included a clearer reference in line 166-167 to the schematic illustration of the study design in S1 Fig of the appendix, as well as the reference to the study protocol in line 176-177, which we also added to the supplementary material in S1 Protocol.*

10) Line 144-146: Method: It would be better for the authors to provide a more detailed explanations regarding the methodology used to standardize various scales into a unified scoring system ranging from 1 to 100.

***Author response:*** *We appreciate your advice and interest in details of the PRO Composite Measure (PRO-CM). We have already discussed the level of detail in the description of the composite a lot internally. The development of the composite was complex and the basis for an individual research work, as we tested various different methods for their quality and validity in order to obtain an index that simplifies the complex data without losing information. We put particular focus on transparency during development and discussed all methods in detail. For this manuscript, however, we decided that this level of detail would distort the focus of the study. Accordingly, we only provide the reader with brief information on the selected methods and refer for more details to our published study on the development of the PRO-CM (see Schöner et al. [3]). Your comment, however, made us realize that an additional sentence to the chosen development methodology will not distort the focus but rather contribute to a better understanding of the index. Therefore, we provided further brief descriptions with details to the development in the manuscript in line 196-198.*

11.) Line 182, Method: It would be better to include a succinct explanation of the 'within-trial cost-effectiveness approach' used in their study, or at least, cite a reference for readers seeking a more comprehensive understanding of this methodology.

***Author response:*** *We use the term within-trial cost-effectiveness analysis as a type of economic evaluation conducted alongside the RCT to assess the cost-effectiveness of the intervention within the trial itself. Therefore, within-trial CEAs directly analyse the costs and outcomes observed from the participants and during the trial duration. To make this point clear we included an additional explanation in the main text in line 237-239.*

12) Line 188-190, Method: A detailed explanation would be beneficial regarding the impact of a 95% winsorisation on the cost data and the rationale behind choosing this specific threshold. Considering that costs are the primary outcome of interest, replacing extreme values with thresholds could notably influence the results. An elaboration on why a 95% winsorisation was preferred over, say, a 99% threshold, would enhance the reader's understanding of the methodological choices.

***Author response:*** *Thank you. We included more information on the methodological choices in the main body of the text. The adjustments can be found in line 244-249. Additionally, the reader finds detailed tables on the cost data per cost category in S/ Table and S8 Table in the appendix. Here, we provided the exact cost values before and after the winsorisation. We chose to winsorise as our cost data, as often observed in cost-data is right-skewed and very long-tailed. I.e. we found extreme outliers by few observations that had remarkably immense costs. This led to very large distortions of the mean and standard deviations and, hence, to wrong findings and conclusions. The choice of a 95% winsorisation is based on a thorough assessment of the data structure. While a 99% winsorisation might seem more conservative in terms of outlier treatment, it did not suffice to mitigate the problem of the distortion of means and standard deviations. However, by applying a 95% winsorisation, we were able to significantly improve this problem and calculate meaningful results without having to make a large cut into the data while still adequately taking into account the effect of outliers. After winsorisation we could reduce the standard deviations and draw more precise conclusions based on robust statistical analyses without excessively distorting the distribution or sacrificing valuable data points.*

**Reviewer #2:**

Overall, this is a well written manuscript on a very important area. The study design and methods that are used are robust. There is a minor concern regarding the data analytical methods that were used, particularly mixed-models. While the investigators used clustering effect of hospitals in modeling, it is not clear how the temporal effect on outcomes/cost was taken into account. In the revised version, the authors should provide revised analytical approach taking in account the time effect.

***Author response:*** *Thank you very much for your positive feedback and the advise, we really appreciate the statistical input. We agree that it would be beneficial and statistically more sound to take into account the temporal effect. Unfortunately, we were unable to include the effect of time in our analysis. This has mainly two reasons. First, the overall study duration was only one year (from hospital admission until 12-month follow-up), hence, the within-trial cost-effectiveness analysis is limited to the period of one year. Second, we couldn’t consider temporal effects within the one-year post-surgery period. Even though in theory we could consider time effects for the intervention group for the 1-, 3- and 6-month follow up, this data is missing in the control group as per study design. As our analysis examines the effect of this intervention on both groups, we were only able to include data in our analysis that was present for both groups, including a time variable. Further research is needed with larger time horizon to more closely examine long term effects of the PROM intervention including potential time effects. Finally, it is to mention, that we examined potential statistical models including an indicator variable of the year of surgery. However, since this variable did not show any statistical relevance in the model, we decided to leave it out. We apologize that we cannot address your suggestion due to the data situation.*

**Reviewer #3:**

1) A novel trial idea, and patient-centred outcome for which the authors should be commended.

***Author response:*** *Thank you very much, we gladly accept the commendation!*

2) Although the authors stated they followed CONSORT for reporting, I could not find a copy of the CONSORT checklist attached with page numbers referencing each point in the manuscript. This would be extremely helpful in ensuring all relevant checklist points about trial conduct were addressed and described.

***Author response:*** *Thank you, that is true. For the initial submission it was not required to upload the corresponding CONSORT checklist. You will find the checklist now in S1 Checklist of the attachments of the revised manuscript. In the text we refer to the CONSORT checklist in line 153-154.*

3) The choice of trial outcome is unusual and somewhat hard to follow. First a composite PROM index was constructed (without validation), and this was combined with costs for an incremental cost-effectiveness ratio. An effect size of (0.15 standard deviation) from the mean in one of the PROMs, or the composite was used, according to the Appendix in the protocol paper. There was no effect size given for costs, or for cost-effectiveness.

***Author response:*** *Thank you very much. First, we would like to address you point regarding the composite measure, referring to our response to comment 10) of Reviewer #1: We have already discussed the level of detail in the description of the composite a lot internally. The development of the composite was complex and the basis for an individual research work, as we tested various different methods for their quality and validity in order to obtain a robust index that simplifies the complex data without losing information. We put particular focus on transparency during development and discussed all methods in detail. For this manuscript, however, we decided that this level of detail would distort the focus of the study. Accordingly, we only provide the reader with brief information on the selected methods and refer for more details to our published study on the development of the PRO-CM (see Schöner et al. [3]). Your comment, however, made us realize that an additional sentence to the chosen development methodology will not distort the focus but rather contribute to a better understanding of the index. Therefore, we provided further brief descriptions with details to the development in the manuscript in line 196-198.*

*Regarding the effect size, it is correct that the effect size of 0.15 SD is assumed for the PRO composite measure in the protocol paper. However, this statement of the study protocol is based on the statistical analysis plan where the effect size rather refers to the outcome-over-cost ratio, hence, includes both outcomes and costs. It was not possible to validly estimate in advance an intervention effect on outcomes or costs due to a lack of comparable data from the literature. Therefore, rather conservative assumptions were made with an effect size of 0.15 SD for the PROM composite and the costs (see also response to comment 1) in Editorial comments). To also enhance the comprehensibility to the reader, we have integrated a supplementary comment into the text referring to the power calculation in the appendix in line 220-221.*

4) Could the authors justify why they chose not to have a primary end point of the trial as a minimally important difference in the PRO, and a secondary outcome of cost-effectiveness? E.g. with an ICER of cost per additional person achieving a meaningful PRO improvement. The primary outcome is not well articulated, and a willingness to pay threshold for the German payer was not provided.

***Author response:*** *As per study protocol and statistical analysis plan the primary outcome of this study was the incremental cost-effectiveness ratio to assess the effect of the intervention on both health outcomes and healthcare expenditures. The statistical analysis plan precisely specifies that the outcome side is given by a PRO Composite Measure. This was developed and validated in a separate study that is cited in the manuscript. We agree that for the assessment of clinical relevance MCIDs play an essential role and should be considered in these types of analyses. Unfortunately, at the time of designing the study no existing MCIDs were available for our study setting. Only at a later stage of the study, we calculated specific MCIDs within the course of the study based on the PROMs used. The results are published in Langenberger et al. [4]. Further research articles that are based on the PROMoting Quality data set will increase the focus on these MCIDs. However, in the present article the focus is on the primary outcome as per study protocol, i.e. the cost-effectiveness.*

*Unfortunately, we were also not provided with willingness-to-pay thresholds from insurers that determine a certain value for cost-effectiveness. Therefore, we calculated the probability of cost-effectiveness, defined as the bootstrapped estimates that have negative incremental costs. Our initial idea was to include a figure with the cost-effectiveness-acceptability curve that assesses the probability of acceptance of the incremental costs for one unit more outcome considering various different WTP-thresholds. Since our results reveal a dominant strategy with better health outcomes and fewer costs, this way of data presentation seemed rather unreasonable as the probability to accept will be 100% for all thresholds. Therefore, we calculated the probability of cost-effectiveness, defined as the bootstrapped estimates that have negative incremental costs. Since our results reveal a dominant strategy with better health outcomes and fewer costs, we consider that problem rather negligible.*

5) Trial and cost-effectiveness sample size estimation. Could the authors please outline the effect size for PRO-CM, effect size for costs, and overall effect size for cost-effectiveness they were testing? It is unclear from Appendix II.

***Author response:*** *Thank you for pointing that out. We kindly refer to our response to comment 1) of the editorial comments and the response to your comment 3): We realize that the details of the preceding power calculation can easily be overlooked. In the body text of the manuscript, we refer to the statistical analysis plan, which can be found in the appendix. The a priori power calculation is listed there in detail. In order to improve the reading flow of the main manuscript, we have decided to move the details to the appendix. Nevertheless, in the following we would like to go into more detail about the a priori power calculation and the set effect size:*

- *Effect size: The effect size was set based on rather conservative assumptions as, firstly, valid estimations for an intervention effect were not possible in advance due to a lack of similar studies with a PROM composite measure and cost incurred for hip and knee replacement patients. Secondly, we assumed the effect from the PROM-based monitoring and alert system to be relatively low against the background of the comparatively limited intervention at patient level – in the intervention group within the one-year follow-up period, only three more PROM surveys were scheduled than in the control group. Further actions then only took place depending on (abnormal) results of the PROM survey. Therefore we assumed an intervention effect size of 0.15 standard deviations.*
- *Power of intervention: We assumed a priori a sufficiently high probability (power of 80%) for the statistical validity of an intervention effect with the common error probability of p = 5%. Assuming this effect size and power of the intervention on the "outcome over cost ratio", the minimum number of study participants required was determined to be n = 1396.*

*We hope that we could sufficiently illustrate our a priori power calculation with this description. To also enhance the comprehensibility to the reader, we have integrated a supplementary comment into the text in line 220-221 referring to the power calculation in the appendix.*

6) Please provide the trial registration number on e.g. Clinicaltrials.govdatabase or similar

***Author response:*** *The PROMoting Quality trial is registered in the German Register for Clinical Studies (DRKS) under registration number DRKS00019916. We included that information in the manuscript in line 149-152 as well as in the last line of the abstract (line 60).*

7) Why was the included sample in this analysis so low, one sixth of the whole randomised population?

***Author response:*** *While the outcome data was available for the whole population, cost data was only available under two conditions: The health economic evaluation was possible only for patients that a) were members in one of the 24 participating statutory health insurances (out of ca. 100 in total), i.e. BARMER and 23 of the BKK Dachverband (the federal association of company health insurances) and b) gave informed consent on processing and analysing their data. Out of the total randomised population of 6807 patients these requirements were met for 1038 patients. Nevertheless, we consider this a decent sample size which suffices to draw statistically valid conclusions. We included additional comparisons of the analysed sample to the full sample that can be found in S9 Table in the supplementary material and are discussed in line 398-405 of the manuscript.*

8) If participation was limited to single health insurer. How were these patients different or similar to the whole trial population in terms of SES, age, sex, region, income, occupation etc. Very large potential for selection bias.

***Author response:*** *The population in the current research was limited to patients that are members of the participating health insurers. However, we had a total of 24 different insurers that together cover approximately 23% of the whole German population and, hence, provide an insightful average profile of the German insured population. For more details on the differences to the whole study population we kindly refer to our response to comment 2) and 3) of the editorial comments. Additionally, we refer to our published secondary analysis of the outcomes, that is based on the whole study population and included more details about differences to the whole sample in the revised manuscript. We also included an additional table (S9 Table) in the supplementary material that shows the descriptive statistics of the whole study population. Our adjustments in the manuscript can be found in line 398-405. We can see some slight differences to the whole population, mainly in the distribution of gender, which we transparently point out in the discussion.*

9) Were the PRO results adjusted for baseline values in the between group comparisons?

***Author response:*** *For our analysis we strictly followed the CONSORT guidelines. Accordingly, it is not intended for RCTs to control for baseline differences, as randomization should balance out any baseline differences. If differences do exist, they are due to pure chance. Nevertheless, we have listed all baseline scores of the PROMs in Table 2 of the manuscript and tested them for statistically significant differences in a separate analysis. Thereby, no significant differences were found. In addition, our primary effect parameter, the PRO-CM, includes the difference between baseline and 12-month follow-up. Thus, the baseline differences were also indirectly taken into account.*

10) The results indicate the intervention worked in hips but not knees. Could the authors re-phrase their interpretation in line with the results, and comment on why this may be the case.

***Author response:*** *Thank you. Our results indeed indicate that the intervention is particularly cost-effective for hip patients, while being less cost-effective for knee patients. We see several possible explanations for this. First, knee patients show more comorbidities and higher BMIs. Therefore, we can assume smaller effectiveness for more complex cases. Also, knee patients already generally receive closer post-surgery attention than hip patients. This could diminish the “being taken care of” effect. Lastly, knee replacements are more complex than hip replacements and require a significantly longer recovery period. Therefore, it might be plausible, that the chosen post-surgery period of 12 month is too short for knee patients. Longer follow-ups with more PROM surveys might enable different insights and more significant effects. However, this is subject to further research on that topic. We adjusted the section in the discussion from line 422-437 in order to address your suggestion and provided more detailed explanations on the low effectiveness for knee replacement patients.*

11) In economic jargon, a dominant or dominated intervention is not usually reported as an ICER, because negative ratios can be difficult to interpret

***Author response:*** *Thank you very much for that comment – we agree that a negative ratio is hard to interpret and can be misleading. We were already discussing on whether or not include the ICER in the manuscript before the submission. To avoid wrong conclusions, we decided to delete the ICER results from the abstract but leave it in the main text of the manuscript in order to provide the complete information because the ICER is the most important and common indicator in cost-effectiveness analyses. However, we added a disclaimer to interpret the negative ratios with caution in line 351-352.*

References

**1**.	Langenberger B, Steinbeck V, Schöner L, Busse R, Pross C, Kuklinski D. Exploring treatment effect heterogeneity of a PROMs alert intervention in knee and hip arthroplasty patients: A causal forest application. Computers in Biology and Medicine. 2023; 163:107118. Epub 2023/06/22. doi: 10.1016/j.compbiomed.2023.107118 PMID: 37392619.

**2**.	Steinbeck V, Langenberger B, Schöner L, Wittich L, Klauser W, Mayer M, et al. Electronic Patient-Reported Outcome Monitoring to Improve Quality of Life After Joint Replacement: Secondary Analysis of a Randomized Clinical Trial. JAMA network open. 2023; 6:e2331301. Epub 2023/09/05. doi: 10.1001/jamanetworkopen.2023.31301 PMID: 37656459.

**3**.	Schöner L, Kuklinski D, Geissler A, Busse R, Pross C. A composite measure for patient-reported outcomes in orthopedic care: design principles and validity checks. Qual Life Res. 2023:1–11. Epub 2023/03/24. doi: 10.1007/s11136-023-03395-0 PMID: 36964454.

**4**.	Langenberger B, Schrednitzki D, Halder AM, Busse R, Pross CM. Predicting whether patients will achieve minimal clinically important differences following hip or knee arthroplasty. Bone & joint research. 2023; 12:512–21. Epub 2023/09/01. doi: 10.1302/2046-3758.129.BJR-2023-0070.R2 PMID: 37652447.
