# Supplementary material for: Cost-effectiveness of a patient-reported outcome-based remote monitoring and alert intervention for early detection of critical recovery after joint replacement: A randomised controlled trial
Source: PLoS Med. 2024 Oct 9;21(10):e1004459. doi: 10.1371/journal.pmed.1004459 (PMC11463742; doi:10.1371/journal.pmed.1004459)
Supplement: Attachment — Submitted filename: 20240717_revisions_response.docx [file pmed.1004459.s021.docx]

Dear editor, dear reviewers,

Thank you very much again for your valuable comments. They have helped us to substantially improve the article. We hope to have included your feedback to your satisfaction. In this document we respond to each of the reviewers´ feedback. Below the reviewers´ comments, we provide our response in blue and describe how we chose to address the feedback. Line references in the responses refer to the marked-up copy of the manuscript.

Please let us know if you have additional feedback or comments. Thank you again for your help in making this a better research contribution.

Yours sincerely,

Lukas Schöner

**Reviewer #1:** The authors have adequately answered all my questions and questions from other reviewers, and have revised the paper accordingly. In my opinion, this paper is acceptable in its current form.

***Author response:*** *Thank you very much for this positive feedback. We are very pleased that our research has aroused your interest and appreciate your thorough review very much.*

**Reviewer #4**

Thank you to the authors for submitting this paper on an interesting topic. I have concentrated on the statistical and health economics aspect of the study, hopefully some of the other reviewers can comment on the intervention and clinical context of the study. I have several major comments, most of which seem to have been picked up by the previous reviewers.

1) PRO-CM

I've simply not convinced by the use of the PRO-CM as the primary outcome measure. Combining so many different measures together and then standardizing them creates something that in my opinion is essentially meaningless. What does a unit change in this PRO-CM actually mean? I know that 0.15 SD change seems to have been quoted in the protocol, but this seems to be completely arbitrary. I would strongly suggest using another measure (either a generic preference-based measure such as the EQ-5D-5L or a condition-specific measure) as the primary outcome and having this composite measure as an experimental secondary outcome. To me there is simply not enough precedent to use this in the ICER and present it as the primary economic outcome.

***Author response:*** *Thank you very much for this helpful and important comment. We are very aware that our primary outcome, the composite measure PRO-CM, is an unfamiliar and somewhat complex construct and that its use may initially be met with skepticism. We are therefore grateful about your feedback and appreciate your suggestion very much. We are convinced that the manuscript can benefit greatly from additional analyses. In the following, we would like to propose a compromise that includes both your suggestion and our initial approach. Further, we would like to point out the background and rationale of using the composite measure PRO-CM and convince you of the added value of its application.*

1. *Compromise: A balanced approach
   To adhere to the study protocol, we would like to continue using the PRO-CM as the primary outcome parameter. However, since it makes perfect sense to conduct further analyses for the purposes of interpretability and robustness checks, we conducted two additional CEAs applying well-known and validated PROMs. The first analysis uses the generic EQ-5D-5L as the outcome, the second the disease-specific HOOS-PS or KOOS-PS, respectively. We see that the results of these additional analyses are in line with the prior results as described in the manuscript. For HR patients considering both the EQ-5D-5L and the HOOS-PS as primary outcomes reveals a dominant intervention with more than 99% of the bootstrapped estimates being located in the south-east (i.e. dominant) quadrant. In KR patients the intervention shows only low effectiveness for the EQ-5D-5L and no effect on KOOS-PS, which is also in line with the prior findings (see Fig 2 in the manuscript).*

*This supplementary analysis demonstrates the robustness of our findings across different evaluation frameworks based on the more familiar and validated PROM tools without detracting from the primary focus on the composite measure. However, instead of using these analyses as the primary outcome and considering the PRO-CM in a secondary sensitivity analysis, as suggested by you, we would like to make a “reversed counter-proposal”: In order to follow the study protocol and thus maintain integrity and good scientific practice, we would like to continue to use the PRO-CM as the main analysis, whereas the two additional analyses are to be understood as secondary analyses, which should provide additional interpretative power and proof of robustness. This should provide a comprehensive and understandable picture of the intervention’s cost-effectiveness and at the same time enable a good understanding of the novel outcome parameter PRO-CM.
In the following, we would like to point out the rationale for using the PRO-CM as primary outcome and convince you of the added value of its application.*

2. *Adherence to study protocol
   First of all, we would like to highlight that in the present manuscript we report the primary outcome of the PROMoting Quality trial. By using the composite rather than one of the individual PROMs we adhere to the pre-set study protocol and statistical analysis plan of the PROMoting Quality trial (Kuklinski et al., 2020). We consider this adherence to the study protocol a cornerstone of good research practice, ensuring consistency, reliability, and validity in our findings. This adherence is crucial for maintaining the integrity of our research and the credibility of our results (following the International Council for Harmonisation’s Good Clinical Practice (ICH GCP) guidelines).*
3. *Effect Size
   The PROMoting Quality trial was powered based on the assumption of a 0.15 SD change in the PRO-CM. We are aware that this is a rather conservative assumption that was done due to the lack of comparative interventions in existing literature. This threshold was selected based on established benchmarks in the literature indicating differences of 0.2, 0.5, and 0.8 standard deviations are considered ‘small’, ‘medium’, and ‘large’ effect sizes respectively (Teare et al., 2014). With 0.15 SD, we made a slightly more conservative assumption, as we expected only small effects and wanted to ensure that the study was not underpowered. This assumption was supported by prior research and expert consensus during the planning phase of the trail, making it a robust basis for powering our study.*
4. *Rationale of using the PRO-CM
   The initial decision in the study protocol to use a composite measure rather than an individual PROM was made for several reasons. The objective of the trial was to investigate how the intervention affects the quality of health improvement from the patient's perspective in orthopedic care. In this context, health should be understood as a comprehensive, multidimensional construct that reflects the improvement in an osteoarthritic condition. The selection of PROMs to capture health is based on the ICHOM (International Consortium for Health Outcomes Measurement) Standard Set for Hip and Knee Osteoarthritis, which consists of the EQ-5D-5L, HOOS-PS/KOOS-PS, and an analogue pain scale. This set was refined by adding the PROMIS Depression and Fatigue sets, as studies show that the health outcome of patients that underwent knee or hip replacement surgery is also affected by their mental conditions. The combined set of PROMs should cover all important patient-related aspects of the osteoarthritic condition and the elective TKR and THR procedures: health-related quality of life (HRQoL), physical functionality, pain, and mental condition. Instead of looking separately on the individual PROMs that only capture one aspect of health (for example, as in our case, the HOOS-PS for physical health or the EQ-5D-5L for general HRQoL), the composite reflects a more comprehensive, holistic picture of health that combines the different health domains captured by many individual PROMs in one index. It therefore enables a comprehensive evaluation of the PROMoting Quality trial that considers all relevant health dimensions and only uses one individual index.*
5. *Measures to mitigate lack of transparency
   Since the application of composite measures face critical challenges in terms of transparency, interpretability and loss of information, we dedicated a separate publication on the rationale and empirical design of composite measures (Schöner et al., 2023). The purpose of this was to counteract the known risks of composites and to make the development and underlying decisions as transparent as possible. Different approaches were used to identify the best possible method, which presents the improvement in health status as accurately as possible without sacrificing important information. The exact calculation can therefore be read and understood in detail there. In very few cases where composite measures are used there is such a detailed description of the approach. We are convinced that not only does this ensure transparency and interpretability, it also makes it possible to break down the values of the composite back into its individual parts.*

*Furthermore, in the manuscript we provide detailed mixed effects regression models in which we assess the intervention effect on the PRO-CM and additionally on all individual PROs that are aggregated in the composite measure. With this we aimed to show the contributions of either PRO to the primary outcome parameter PRO-CM and generate a broader understanding of its composition.*

*We hope this explanation clarifies our rationale and underscores the importance of adhering to our study protocol. We believe that this approach ensures the integrity of our research and also aligns with best practices in the field. At the same time, we are very grateful for your feedback and are convinced that we have been able to make significant improvements to the manuscript based on it. We hope that with this compromise we have adequately addressed your comment.* *The adjustments in the marked-up manuscript can be found in the abstract, in the methods section line 264-271 and in the results section line 388-397.*

2) Probability of Cost-Effectiveness

Calculating the “probability of cost effectiveness” from an ICER within a Bayesian cost effectiveness framework explicitly requires a threshold to compare the ICER to. Without a cost effectiveness threshold for the ICER for the PRO-CM, the authors use the proportion of bootstrapped estimates that have negative incremental costs. This is incorrect and should be dropped from the paper as it is misleading. It’s essentially implying the threshold is 0.

***Author response:*** *Thank you very much for this helpful remark.* *It is correct that the present analysis implies a threshold of 0 which indeed is misleading. The first rationale for this is that we have detected a dominant intervention that is cost-effective even under this assumption. It was aimed to provide a further indicator in addition to the ICER that gives an idea of how to interpret the results. Our initial idea was to include a figure with the cost-effectiveness-acceptability curve that assesses the probability of acceptance of the incremental costs for one unit more outcome considering various different WTP-thresholds. Since our results reveal a dominant strategy with better health outcomes and fewer costs, this way of data presentation seemed rather unreasonable as the probability to accept will be 100% for all thresholds. Therefore, we calculated the probability of cost-effectiveness, defined as the bootstrapped estimates that have negative incremental costs. However, we fully agree that this is misleading and decided to drop this part from the manuscript. We decided to exchange the metric “probability of cost-effectiveness” for the “probability of dominance” which we define as share of bootstrapped point estimates that fall into the dominant quadrant.* *Find the adjustment in the marked-up manuscript in line 254-257 and the updated values in the result section.*

3) Dominant and Dominated Interventions

As picked up by the reviewers, ICERs for dominant and dominated interventions don’t have any meaning and should be removed from all parts of the paper, not just the abstract.

***Author response:*** *Thank you very much for that comment. We agree that the ICER for dominant and dominated interventions are hard to interpret and can be misleading. We still considered it an important indicator that is found in most CEAs and therefore left it in the main body of the manuscript to give the complete picture. However, based on your feedback we decided to remove it entirely to avoid misleading information and only mention in the manuscript that with negative incremental costs and positive incremental effects it is meaningless to calculate and interpret the ICER. The corresponding adjustments in the marked-up manuscript can be found in line 351-375.*

4) Controlling for Baseline PRO-CM

As picked up by one of the other reviewers, adjusting for baseline PRO-CM could be a good idea. Given the nature of the results and the intervention, I would like to see a sensitivity analysis with baseline PRO-CM included in the mixed effect regression models. This has been good practice in within-trial economic evaluations for around 20 years, please see Manca, Hawkins & Sculphur (2004): <https://doi>.org/10.1002/hec.944

***Author response:*** *Thank you very much for this advice and providing us with the relevant literature on this topic. We agree with you that it could provide further insights to include an additional sensitivity analysis in which we control for the baseline scores. Therefore, we added sensitivity analyses to the manuscript and included corresponding plots of the estimates in the appendix. We found similar patterns as in the original regression. The adjustments in the marked-up manuscript can be found in line 228-229 and 325-329.*

5) QALYs/CUA

As the authors collected EQ-5D-5L, I’m confused why they didn’t consider presenting a CUA alongside the CEA?

***Author response:*** *Thank you for your valuable feedback. We appreciate your suggestion to present a cost-utility analysis alongside the cost-effectiveness analysis using the EQ-5D-5L to calculate quality-adjusted life years (QALYs). In the following, we would like to address several points regarding our decision to focus solely on the CEA.*

*As we also described in our response to your comment 1, by performing a CEA instead of a CUA we adhere to the previously defined study protocol. In this manuscript we report the primary outcome of the PROMoting Quality trial, which is a CEA with the PRO-CM as outcome parameter. Performing a CUA based on QALYs was not intended for the evaluation of the trial.*

*This had several reasons. Due to their ethical and methodological problems, QALYs do not play a significant role in health economic evaluations and decision-making in the German context. The Institute for Quality and Efficiency in Health Care (IQWiG) expresses various doubts for applying QALYs in health economic analyses and criticizes the underlying ethical, legal, cultural and methodological assumptions (IQWiG, 2023). Further there is no defined threshold to guide policy decisions based on QALYs. The IQWiG emphasizes other measures of health benefit, in particular patient-reported outcomes to measure different health dimensions from the patient’s perspective. In conclusion, the IQWiG advises against the use of QALYs in a health economic context. Consequently, presenting a CUA might not align well with the prevailing evaluation standards and practices in Germany.*

*We can look at a comprehensive set of PROMs that measure patient benefit in terms of health-related quality of life, physical health and mental health. In our view, this provides an even more accurate and holistic picture of the quality of treatment and effectiveness of the intervention, which would not be possible with QALYs. Now that we have, based on your helpful comment 1, in addition to the main analysis with the PRO-CM, two further CEAs with the generic EQ-5D-5L and the disease-specific HOOS-PS and KOOS-PS, we are convinced that we can show very informative and valuable results that would not be further enhanced by a CUA.*

**References**

IQWiG. (2023). *Allgemeine Methoden Version 7.0* (7.0). Institut für Qualität und Wirtschaftlichkeit im Gesundheitswesen (IQWiG).

Kuklinski, D., Oschmann, L., Pross, C., Busse, R., & Geissler, A. (2020). The use of digitally collected patient-reported outcome measures for newly operated patients with total knee and hip replacements to improve post-treatment recovery: Study protocol for a randomized controlled trial. *Trials*, *21*(1), 322. https://doi.org/10.1186/s13063-020-04252-y

Schöner, L., Kuklinski, D., Geissler, A., Busse, R., & Pross, C. (2023). A composite measure for patient-reported outcomes in orthopedic care: Design principles and validity checks. *Quality of Life Research*, *32*(8), 2341–2351. https://doi.org/10.1007/s11136-023-03395-0

Teare, M. D., Dimairo, M., Shephard, N., Hayman, A., Whitehead, A., & Walters, S. J. (2014). Sample size requirements to estimate key design parameters from external pilot randomised controlled trials: A simulation study. *Trials*, *15*(1), 264. https://doi.org/10.1186/1745-6215-15-264
