# Supplementary material for: Cost-effectiveness of a patient-reported outcome-based remote monitoring and alert intervention for early detection of critical recovery after joint replacement: A randomised controlled trial
Source: PLoS Med. 2024 Oct 9;21(10):e1004459. doi: 10.1371/journal.pmed.1004459 (PMC11463742; doi:10.1371/journal.pmed.1004459)
Supplement: Attachment — Submitted filename: 20240808_revisions_response.docx [file pmed.1004459.s022.docx]

Dear editor, dear reviewers,

Thank you very much for your message. We are very glad to hear that you intend to accept the paper for publication in PLOS Medicine. Also thank you for your valuable comments and remarks in the manuscript. They have helped us to substantially improve the article. We hope to have included your feedback to your satisfaction. In this document we respond to each of the editors’ and reviewers´ feedback. Below the comments, we provide our response in blue and describe how we addressed the feedback. Line references in the responses refer to the marked-up copy of the manuscript.

Thank you again for your help in making this a better research contribution.

Yours sincerely,

Lukas Schöner

**Requests from Editors:**

**General**

We feel that the manuscript could be improved with respect to structure and clarity. When revising, please keep in mind that the manuscript should be accessible to readers who may not be familiar with the topic.

***Author response:*** *We thoroughly reviewed the entire manuscript to identify areas where the structure could be improved and the clarity enhanced. Your helpful comments greatly contributed to our ability to significantly refine the manuscript. For instance, we have now made a clearer distinction between the inclusion and exclusion criteria and emphasized the differences between the intervention and control groups by restructuring the Procedures section. Additionally, we revised the wording in many sections to avoid confusion and misinterpretation. For example, based on your feedback, we clarified the primary outcome of the study (i.e., cost-effectiveness) and the main effect parameter (PRO-CM). Other details, such as a brief description of the 'Standard of Care' and specifics of the statistical analysis plan, help ensure that relevant background knowledge is provided and that the manuscript's clarity is enhanced. We are confident that, based on your comments, we have significantly improved the manuscript's clarity and structure.*

We suggest removing the abbreviations "KR" and "HR" and spelling them out throughout the manuscript.

***Author response:*** *We removed the abbreviations “KR and “HR” throughout the manuscript and used a more patient-centered wording instead.*

PLOS Medicine prefers the use of patient-centered language, e.g. patients undergoing hip replacement and patients undergoing knee replacement (or similar). Please revise throughout the manuscript, including tables and figures (including those in the Supporting Information).

***Author response:*** *Thank you for this fair comment. To increase patient-centered language we first removed the abbreviations “KR and “HR” throughout the manuscript (including figures and tables) in accordance with your prior comment. Further, instead of using the term hip or knee patients we followed your suggestion and changed the wording to “patients with hip replacement” and “patients with knee replacement” throughout the manuscript.*

We have noticed that you refer to results that are not statistically significant as insignificant. Please note that insignificant usually implies unimportance, without statistical connotations. Please revise and change to 'non-significant' throughout.

***Author response:*** *Thank you for pointing that out. We have corrected this and now only use the term non-significant.*

**Data availability**

The Data Availability Statement (DAS) requires revision. Please include an appropriate contact (web or email address) for inquiries (this cannot be a study author).

***Author response:*** *Thank you. We revised the DAS accordingly by adding more information, including the contact details of the Data Protection Officer at TU Berlin who can be contacted in case of inquiries for data access. Revisions made in line 574-580.*

**Abstract**

1) Please combine the Methods and Findings sections into one section.

***Author response:*** *Amendments made*

2) l.39: “3697 hip and 3110 knee replacement patients” - We prefer the use of patient-centered language (i.e. “3697 patients with hip replacement and 3110 patients with knee replacement”). Please revise accordingly throughout the Abstract and the main text.

***Author response:*** *Thank you, amendments made. Instead of using the term hip or knee replacement patients we followed your suggestion and changed the wording to patients with hip replacement and patients with knee replacement throughout the manuscript.*

3) l.41: Please add details about the randomization, e.g. 1:1.

***Author response:*** *Amendments made.*

4) l.46ff: Per CONSORT, please note that only the primary outcome of the trial should be reported in your Abstract. Secondary outcomes should only be included in the Abstract if all secondary outcomes are fully reported. For trials that have many secondary outcomes, the Abstract should be limited to reporting the primary outcome.

***Author response:*** *Thank you. We removed statements referring to secondary outcomes from the abstract and report only the primary outcome.*

5) l.47ff: Please define abbreviations at first use, such as HOOS-PS, KOOS-PS or CI.

***Author response:*** *Thank you. Based on your prior comment we decided to drop statements about secondary outcomes. Therefore, the abbreviations of the PROMs are not longer used in the abstract. However, we defined all remaining abbreviations, including CI, at first use.*

6) In the last sentence of the Abstract Methods and Findings section, please describe the main limitation(s) of the study's methodology.

***Author response:*** *Thank you. We added a description of the main limitation to the abstract. Revisions made in line 55-56.*

7) ll.56-57: The term "tendency" is used to refer to a nonsignificant P value, please remove and revise the conclusions accordingly.

***Author response:*** *Thanks for that advise. We agree that the wording is misleading. We changed “tendency” to “weakly significant cost reduction”. In accordance with your similar comments on the term “tendency” and “trend” we changed it accordingly throughout the manuscript.*

8) Please remove the Funding Statement from the Abstract.

***Author response:*** *Amendments made.*

**Author Summary**

1) Please see the comments regarding patient-centered language and the term ‘tendency’ under ABSTRACT and revise accordingly.

***Author response:*** *Thank you. We revised the author summary regarding a more patient-centered language in line with your prior general comments. Further we changed “tendency” to “cost reduction”.*

2) In the final bullet point of ‘What Do These Findings Mean?’ Please include the main limitations of the study in non-technical language.

***Author response:*** *Thank you. We added the main limitation in a more non-technical language.*

**Introduction**

1) l.105: Please define OECD.

***Author response:*** *Amendments made.*

2) ll.115-116, please change to: In further oncological studies, it was shown that the intervention was cost-effective [17,18].

***Author response:*** *Amendments made.*

**Methods and Results**

1) ll.138-149: Please outline the main inclusion/exclusion criteria in the main text. You may then refer to the detailed criteria in the Supplementary Material and the published protocol.

***Author response:*** *Thank you. We changed and restructured the text accordingly and added further details to emphasize the inclusion and exclusion criteria. Revisions made in line 144-150.*

2) l.162: “Patients in the control group received the standard of care.” – We suggest a brief outline of what SOC entails, as a global audience may have different experiences with SOC after hip and knee replacement surgery.

***Author response:*** *Thank you for that suggestion. We added a brief description of what is included in Standard of Care in order to provide a good understanding for a global readership. Revisions made in line 181-186.*

3) ll.160-172: We suggest revising this paragraph for clarity and structure.

***Author response:*** *Thank you for your feedback. We agree that the section could benefit from a better structure. We have rearranged the text accordingly so that there is now a clearer distinction between the treatments in the intervention and control groups. First we explain the exact details of the intervention group and then the details of the control group. In our view, this has created a clearer structure and better understanding of the procedures. Revisions made in line 168-188.*

4) l.193: Please define ‘SD’ at first use.

***Author response:*** *Amendments made in line 211.*

5) l.214: Please provide details on the power calculation and the statistical analysis plan in the main text.

***Author response:*** *Thank you. We added further details on the power calculation and statistical analysis plan of the study. Revisions made in line 232-237.*

6) l.224: The terms gender and sex are not interchangeable (as discussed in https://www.who.int/health-topics/gender); please use the appropriate term.

***Author response:*** *Thank you. We changed the term “gender” to “sex” throughout the entire manuscript.*

7) l.225: Please define ‘BMI’ at first use.

***Author response:*** *Thank you. Amendments made in line 248.*

8) l.232: Please define ‘CI’ at first use.

***Author response:*** *Thank you. Amendments made in line 255.*

9) l.259, please change to: “We focused… that only differed…”

***Author response:*** *Thank you. Amendments made.*

10) Table 1: Please define ‘PROM’.

***Author response:*** *Thank you. Amendments made.*

11) l.265ff: Please revise for tense. Also, please temper claims of primacy of results by stating, "to our knowledge" or something similar (i.e. Since the application of the PRO-CM as primary outcome parameter was, to our knowledge, novel and…).

***Author response:*** *Thank you. We revised the result and discussion section for tense and tempered the corresponding statements following your suggestion.*

12) l.267: Please define ‘CEA’ at first use.

***Author response:*** *Thank you. Amendments made in line 203-204.*

13) We feel that it may be confusing to readers as to what the primary outcome of the study is. In lines 186-187 you state that you are using a PRO composite measure (PRO-CM) as the primary outcome, which does not mention the cost-effectiveness component, while in line 249 you specify the study's main outcome, i.e., the incremental cost-effectiveness ratio (ICER). We suggest revising the manuscript to improve clarity and suggest that under "Outcomes" you first clearly state the primary outcome, and the secondary outcomes followed by a detailed description of these (as already done).

***Author response:*** *Thank you very much for pointing out this misleading wording. We have made several changes in the Outcomes section so that the difference between the PRO-CM and the primary outcome of the study is now clearer. While the “primary outcome” is the cost-effectiveness (ICER) of the intervention, we define PRO-CM as “main effect parameter” of the evaluation. We changed the terminology throughout the manuscript. We hope that this sufficiently clarifies the primary outcome of the study and counteracts potential confusion. However, we would like to stick to the basic structure as it is because we believe that it is important to first introduce the individual PROMs before explaining the PRO-CM concept. We hope that we were still able to adequately address your comment with our adjustments in the text. Revisions made in line 190-205.*

14) Figure 1: Please define ‘PROM’. Please revise with regard to the use of patient-centered language. Also, please change “patients excluded without consent or with non-participating health insurance” to “patients excluded due to lack of consent or non-participating health insurance”.

***Author response:*** *Thank you. We made the corresponding modifications in Figure 1 and added the definition of PROM in the figure legend.*

15) l.285: Please remove the word ‘respectively’.

***Author response:*** *Thank you. Amendments made.*

16) l.286: In brackets, please add how ‘obese’ was defined.

***Author response:*** *Thank you. We added the BMI threshold for obesity in brackets.*

17) ll.285-286, please change to: 32.8% of patients undergoing hip replacement and 53.1% of patients undergoing knee replacement were obese.

***Author response:*** *Thank you for the suggestion. We made the amendment.*

18) ll.286-287, please change to: 38.4% of the patients who underwent hip replacement surgery, but only 29.0% of the patients who underwent knee replacement surgery had no comorbidities recorded.

***Author response:*** *Thank you. Amendments made.*

19) Table 2: Please exchange ‘gender’ with ‘sex’.

***Author response:*** *Thank you. We changed the term “gender” to “sex” throughout the entire manuscript.*

20) l.291ff: We suggest describing the results throughout as follows: “Comparative analyses showed that, for patients undergoing hip replacement, the PRO-CM [26] was significantly higher…”

***Author response:*** *Thank you for this suggestion. We revised the manuscript accordingly with regard to a more patient-centered language and rather speak about “was higher” then “improved more”.*

21) l.293: Please include statistical information when reporting numerical values (i.e. “51.22 SD (9.66)”).

***Author response:*** *Thank you. Amendments made.*

22) ll.293-294: Please rephrase (The intervention group did not improve, but the outcome measure was significantly higher in the intervention group).

***Author response:*** *Thank you. We slightly adjusted your suggestion and rephrased it to “The intervention group thus had a 5.22% higher PRO-CM than the control group”.*

23) Table 3: Please spell out ‘y’ and ‘Adj’. We also suggest spelling out ‘IG’ and CG’ and adding a definition for ‘Incremental’ below the table.

***Author response:*** *Thank you. Amendments made.*

24) l.296ff: Please revise for tense (These findings were supported…).

***Author response:*** *Thank you for pointing that out. We revised the whole manuscript for tense and made the corresponding corrections.*

25) Figure 2: Please include the relevant abbreviations in the figure description and provide exact p-values instead of asterisks with significance levels. It currently appears as if "Hip/Knee replacement patients" are the y-axis labels. Assuming these serve as headings, please move them above the graph and adjust them using patient-centered language.

***Author response:*** *Thank you for your helpful feedback. We created a new version of the figure. Instead of the asterisks, we have now inserted the exact p-values next to the estimates, placed the graph headers above the individual graphs and used patient-centered language. In addition, we have slightly changed the layout of the graph to a more appealing design using a more neutral color scheme and placing the two graphs next to each other.*

26) l.333: The term "tendency" is used to refer to a nonsignificant P value, please remove and revise accordingly.

***Author response:*** *Thank you. We changed tendency for effect.*

27) ll.342-343: “The mixed effect model, however, only shows a weakly significant intervention effect on the KR costs.” – We suggest adding a reference to Figure 2.

***Author response:*** *Thank you. We included a reference to Figure 2.*

28) Figure 3: Please provide a unit for cost. Please define ‘PROM’.

***Author response:*** *Thank you. We made the corresponding changes (Cost in €) and added the definition of PROM in the caption of Figure 3.*

29) l.371ff: Please revise for tense (e.g. “The findings from our base case models were supported by the sensitivity analyses.”)

***Author response:*** *Thank you. We revised the whole manuscript for tense and made the corresponding corrections.*

30) l.380: The term "trend" is used to refer to a nonsignificant P value. The term trend should be used only when the test for trend has been conducted. Please revise accordingly.

***Author response:*** *Thank you. Amendments made.*

**Discussion**

1) l.408: Please rephrase “Main effects of the intervention for the total sample” to avoid referring to participants as samples. Please revise throughout the main text.

***Author response:*** *Thank you for the feedback. We revised this throughout the manuscript.*

2) l.412ff: The terms gender and sex are not interchangeable (as discussed in https://www.who.int/health-topics/gender); please use the appropriate term and revise throughout.

***Author response:*** *Thank you. We revised the entire manuscript and made corresponding corrections.*

3) ll.430-433: We feel that the statements are repetitive of each other, please revise.

***Author response:*** *Thank you. We rephrased this section to avoid redundancies.*

4) l.452ff: When discussing results, please use past tense.

***Author response:*** *Thank you. Amendments made throughout the manuscript.*

5) l.498, please change to: “study demonstrated”

***Author response:*** *Thank you. Amendments made.*

**References**

Please ensure that journal name abbreviations match those found in the National Center for Biotechnology Information (NCBI) databases (http://www.ncbi.nlm.nih.gov/nlmcatalog/journals), and are appropriately formatted and capitalised. For example, “Patient Related Outcome Measures” in reference [2] should be “Patient Relat Outcome Meas”.

***Author response:*** *Thank you. We double-checked all references to ensure they match the abbreviations in the NCBI database.*

**Supplementary Material**

1) Thank you for providing the completed CONSORT checklist. Please replace the page numbers with paragraph numbers per section (e.g. "Methods, paragraph 1"), since the page numbers of the final published paper may be different from the page numbers in the current manuscript.

***Author response****:* *Thank you.* *We have updated the CONSORT checklist by referring to section names and paragraphs numbers per section instead of page numbers.*

2) In the published article, supporting information files are accessed only through a hyperlink attached to the captions. For this reason, you must list captions at the end of your manuscript file. You may include a caption within the supporting information file itself, as long as that caption is also provided in the manuscript file. Do not submit a separate caption file.

When SI files are contained with a single file:

***Author response****: Does not apply.*

When SI files are uploaded as separate files:

Please label tables as ‘S1 Table’ (so on) and figures as ‘S1 Fig’ (and so on).

Any additional documents (protocols/analysis plans etc.) can be labelled as ‘S1 Protocol’, for example. Please cite items as exactly as labelled.

***Author response****:* *Thank you.* *We have checked all supplemental material again and approved it.*

3) Please revise the Supplementary Material according to the comments above. Please note that the Supplementary Material will be published as provided by the authors.

***Author response****:* *Thank you.* *We have checked all supplemental material believe it meets all requirements. Additionally, we updated the CONSORT checklist.*

**Social Media**

To help us extend the reach of your research, please provide any X (formerly known as Twitter) handle(s) that would be appropriate to tag, including your own, your co-authors’, your institution, funder, or lab. Please enter in the submission form any handles you wish to be included when we post about this paper.

***Author response****:* *Thank you for mentioning that. We will enter our Institution’s X handles in the submission form.*

**Comments from Reviewers**

Reviewer #4: Thank you to the authors for responding so comprehensively to my comments and making the requisite changes to manuscript.

I think the compromise of retaining the primary outcome measure (to be in line with the protocol) but adding the secondary outcomes is fair.

***Author response:*** *Thank you. We are glad to hear that and think that the manuscript highly benefitted from your suggestion.*

I think the justification for the 0.15SD is fair, however I would like an extra sentence referencing Teare et al (2014) explaining this in the manuscript before publication.

***Author response:*** *Thank you. We added further details on the rationale and cut-off values of the chosen effect-size and included the corresponding reference of Teare et al (2014). Revisions made in line 232-237.*

I'm still not convinced how useful the "probability of dominance" is as a metric, but don't think it is a major issue as it is just an output from Figure 3. If the authors think they should retain it, then that is fine with me.

***Author response:*** *Thank you, we are glad to hear that you are fine with keeping this metric. We also see it more as a small, however, not quite uninteresting detail that can provide additional insights and would therefore like to report it.*

Thank you for removing the text regarding negative ICERs, and including the extra analysis which includes controlling for baseline EQ-5D-5L.

***Author response:*** *Thank you again for this suggestion. We agree that negative ICERs are misleading. We consider the sensitivity analyses that control for baseline scores an invaluable contribution to the robustness of our results.*

The justification regarding not conducting a CUA is of course correct (my mistake!)

***Author response:*** *Thank you. We are glad we could convince you.*

If the authors add the extra sentence regarding the justification of the 0.15SD effect size, I am happy to approve the paper for publication, and don't need to review it again. Good luck to the authors with this one!

***Author response:*** *Thank you very much for your valuable feedback. Your comments have enabled us to make significant improvements to the manuscript.* *We are very glad to see that you are satisfied with our changes and approve the manuscript for publication.*
